# Understanding Anomalous Cage-Escape Dynamics in Photoredox Processes Driven by a Fe(III) N‑Heterocyclic Carbene Complex

**DOI:** 10.1021/jacs.5c04296

**Published:** 2025-07-30

**Authors:** Iria Bolaño Losada, Ulf Ryde, Petter Persson

**Affiliations:** Division of Computational Chemistry, Department of Chemistry, Lund University, P.O. Box 124, SE-22100 Lund, Sweden

## Abstract

Solvent cage-escape dynamics of bimolecular photoredox products in solution has been investigated computationally through a combination of molecular dynamics simulations and quantum chemical calculations. The present work focuses on the photoinduced oxidation of the organic electron donor dimethylaniline (DMA) by a Fe­(III) N-heterocyclic carbene photosensitizer (Fe­(III)­NHC^+^) in two different solvents, serving as an example of current interest due to their relevance for the development of earth-abundant photocatalytic systems. Calculated solvent cage-escape yields of radical-cation and neutral photoproducts (DMA^•+^ and Fe­(II)­NHC, respectively) by molecular dynamics simulations reveal more favorable solvation in acetonitrile than in dichloromethane following the initial photoinduced charge-separation. These results agree with basic expectations from solvent polarity considerations but give an opposite trend compared to experimentally reported cage-escape yields. Alternative cage-escape mechanisms were therefore considered computationally to account for the anomalous experimental cage-escape yields. Both quantum chemical calculations and molecular dynamics simulations support the formation of radical-cation dimers ( ${\left[{\left(DMA\right)}_{2}\right]}^{•+}$ ), allowing for more efficient charge migration involving the radical-cations as the polarity of the solvent is decreased. The results further demonstrate the ability of the counterion (PF_6_^–^) to stabilize the photoproducts through radical-cation–anion pairing, suggesting that these bimolecular interactions can also play an important role to preferentially promote photoproduct formation in less polar solvents. Both radical-cation dimer formation and radical-cation–counterion interactions are therefore proposed to provide additional pathways that help to explain the experimental observations of anomalous solvation dependence of the cage-escape dynamics in the investigated system. The broader implications of the bimolecular cage-escape processes on photocatalytic reaction dynamics are also considered based on our findings about light-induced intermolecular interactions.

## Graphical abstract

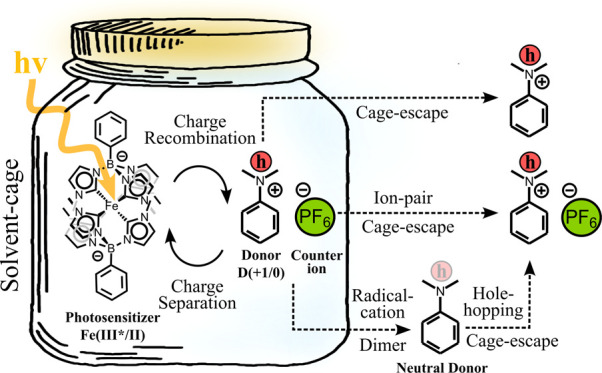

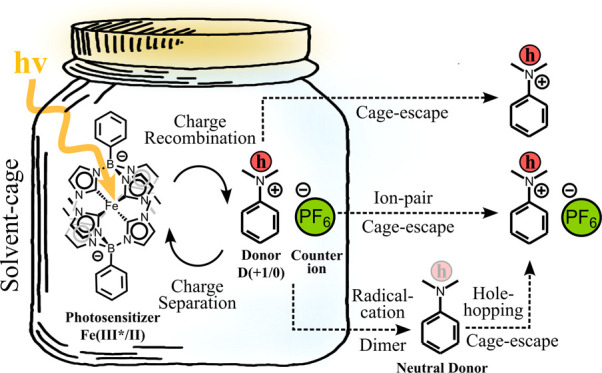

## Introduction

Transition-metal complexes are promising light harvesters for a range of photocatalytic processes.^1–4^ The urgent need to reduce the environmental impact from the use of rare metals together with the urge of cost reduction in the production of devices such as dye-sensitized solar cells (DSSC)^5^ or organic light-emitting diodes (OLEDs)^6^ has motivated extensive studies of earth-abundant transition-metal complexes.^7^ Fe,^8,9^ Co,^10,11^ and Cr^12,13^ are some examples of transition metals that have shown promising photophysical properties for replacing Ru and Ir metal complexes.^14^ The outstanding photostability and excited-state lifetimes of some recent earth-abundant transition-metal complexes with metal-to-ligand charge transfer (MLCT) or ligand-to-metal charge transfer (LMCT) states have broadened their application toward photochemistry.^15–19^

The photoactivity of ^3^MLCT excited states has been widely studied, with a special emphasis on Ru­(II) complexes. In contrast, little is known regarding ^2^LMCT excited states.^20–24^ The ${\left[Fe\left(III\right){\left(phtmeimb\right)}_{2}\right]}^{+}$ (phtmeimb = phenyl­[tris­(3-methylimidazol-1-ylidene)]­borate) complex with N-heterocyclic carbene ligands (Fe­(III)­NHC^+^) featured a long-lived ^2^LMCT state, and the ∼2 ns lifetime of its excited state prompted a study of photoinduced electron transfer with both one-electron donor and one-electron acceptor molecules in acetonitrile (ACN).^25^ The low photoproduct yields observed in the experiments coincided with low calculated cage-escape (CE) yields of only 5%. Further studies in the electron transfer dynamics of the same complex with two well-known (sacrificial) electron donors, triethylamine (TEA) and dimethylaniline (DMA), provided evidence of low photoproduct formation.^26^ Despite efficient charge-separation for both electron donors (with rates of 0.05 ps^–1^ and 1.25 ps^–1^ for TEA and DMA, respectively), charge-recombination was identified as a fast process, with a rate constant for both donor systems of 0.2 ps^–1^.

The study of photoinduced electron transfer for Fe­(III)­NHC^+^ with several electron donors was continued by Aydogan et al.^27^ They observed that the yield of the final photoproduct depended strongly on the choice of solvent, which was attributed to the solvent cage effects. They reported CE yields of ∼60% in dichloromethane (DCM) but only ∼2% in ACN with DMA as the donor, showing that CE can compete with electron back-transfer under certain conditions. Previous studies of charge-recombination events between two *d* ^6^ photosensitizers and a series of anthracene derivatives by Olmsted and Meyer suggested that CE could outcompete the charge-recombination reaction as a result of spin-flip in the spin-forbidden reverse electron transfer reaction.^20^ Therefore, the spin-allowed charge-recombination from reduced Fe­(II)­NHC (neutral singlet) and doublet oxidized donor, D^•+^, to the doublet ground-state Fe­(III)­NHC^+^ and neutral donor may contribute to the fast charge-recombination.^26^ The special charge-pairing in this chemical system, with a positive–neutral molecule pair in both the ground state and the charge-separated state, provides a different electrostatic pairing effect compared to the classical cation–anion charge-separated state with two neutral molecules in the ground state.^28^

Given the anomalous experimental observations in the Fe­(III)­NHC^+^–donor system and the recent attention given to CE mechanisms,^29–32^ it is interesting to consider more thoroughly how the CE dynamics depend on different factors associated with solvation, electrostatics, and bimolecular interactions.^33–36^ In this study, we have therefore investigated the dynamics after charge-separation of Fe­(III)­NHC^+^ and DMA through a combination of quantum chemical calculations and classical molecular dynamics (MD) simulations to better understand the influence of these factors on the peculiar CE dynamics observed in this system. Figure 1 schematically illustrates the CE process targeted in this work. The impact of solvent effects and counterion electrostatics on the slow charge-recombination process was addressed by modeling the Fe­(II)­NHC photosensitizer, the PF_6_^–^ counterion, the DMA^•+^ molecule, and solvent (either ACN or DCM) with a parameterized force field.

**Figure 1:**
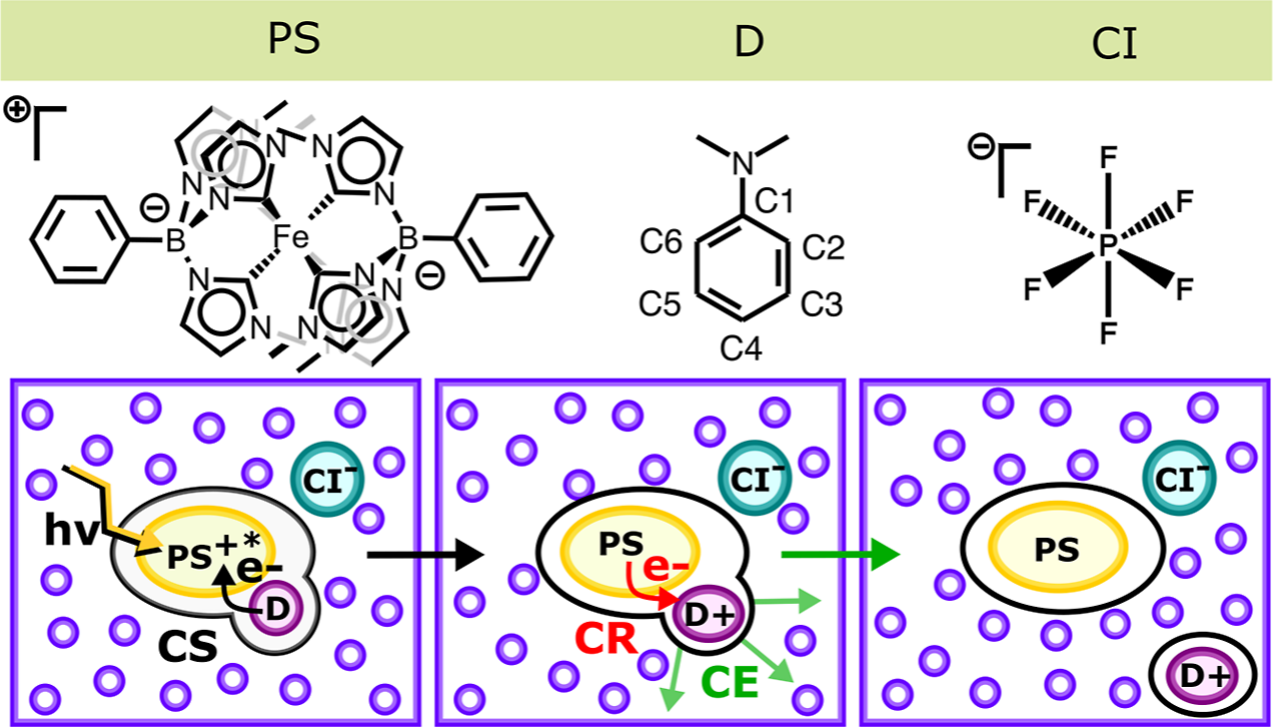
(Top panel) Chemical structures of the photosensitizer (PS), the electron donor (D), and the counterion (CI) considered in this study. Atom numbering for the donor molecule is included. (Bottom panel) Schematic representation of charge-separation (CS), charge-recombination (CR), and cage-escape (CE) steps in the bimolecular interactions between PS and D. The CI provides overall charge neutralization.

## Computational Details

Describing the high complexity of the system involving different electronic states requires adopting a computational strategy that includes certain simplifications. A complete description of this problem by a quantum mechanics (QM) approach is challenging because the charge-separated state is a high-energy state. Neglecting the electronic effects, e.g., charge-recombination, allows us to evaluate isolated aspects such as solvent organization and diffusion processes by molecular mechanics (MM) in both the ground and charge-separated state. Furthermore, using a force field facilitates achieving desirable simulation times on nanosecond time scales, typical in diffusion processes in solution. Certain electronic factors that can affect the competition with the fast charge-recombination in a picosecond time range, for instance, the hole delocalization in electron donor solvents, can be integrated with a QM/MM description. Technical details of the classical and QM/MM molecular dynamics simulations, molecular aggregations, hole delocalization, and electrochemical calculations are included in this section.

### Molecular Dynamics Simulations

The system formed by FeNHC, DMA, and PF_6_^–^ in solution (ACN or DCM) was modeled before and after electron transfer (between FeNHC and DMA) as Fe­(III)­NHC^+^ and DMA, and Fe­(II)­NHC and DMA^•+^, respectively. Fe­(III)­NHC^+^, Fe­(II)­NHC, DMA, DMA^•+^, PF_6_^–^, ACN, and DCM were modeled using MM with parameters from the general AMBER force field (GAFF2) in the AMBER 18 software suite.^37,38^ The good performance of the utilized force field has been validated through comparison with X-ray data and previously reported force field results of the Fe­(III)­NHC^+^ species by Diez-Cabanes et al. (Table SI.1 and Figure SI.1).^25,39^ The tleap module was used to generate all of the input files. All molecules were parameterized by first optimizing each isolated molecule with the B3LYP* ^40,41^ functional and the 6-311G­(d) basis set. The polarizable continuum model (PCM) was applied to model the ACN solvent (ϵ = 35.7) using radii from the UFF force field. Frequencies and electrostatic potentials were calculated at the same level of theory, the latter at points sampled using the Merz–Kollman (MK) scheme with the radius for iron set to 2.0 Å.^42^ These calculations were performed in Gaussian 16.^43^ The potentials were employed to fit atomic charges using the restrained electrostatic potential approach (RESP).^44^ Missing parameters were obtained from the Hessian matrix from the frequency calculation with the Seminario approach.^45,46^ The Fe ion was described by a bonded force field obtained from a frequency calculation of the Fe­(III)­NHC^+^ or Fe­(II)­NHC complex.^46,47^ AMBER files with all nonstandard force field parameters are given in the Supporting Information.

The nonbonded interactions were truncated at 8 Å, and an Ewald summation was used to account for the long-range Coulombic interactions. An initial simulation of Fe­(III)­NHC^+^, DMA, and PF_6_^–^ or Fe­(II)­NHC, DMA^•+^, and PF_6_^–^ was performed in vacuum to generate many possible conformations for the interaction of the three molecules. The system was equilibrated in a three-step fashion: (i) 1000 steps of minimization, (ii) 10 ps MD simulation with a time step of 0.5 fs performed at a constant volume (*NVT* ensemble), and (iii) final MD equilibration of 100 ns with a 2 fs time step in the *NVT* ensemble using the SHAKE algorithm to fix the length of all bonds involving H atoms.^48^ The latter two simulations were performed at 300 K with a Langevin thermostat with a collision frequency of 2 ps^–1^.^49^

Among the resulting trajectories, 50 snapshots were randomly selected as starting structures and were solvated in a previously equilibrated solvent box with a minimum solute-to-edge distance of 20 Å and with periodic boundary conditions (PBC). Each of these 50 models was subjected to 1000 steps of minimization, 10 ps *NVT* MD simulation without SHAKE, 1 ns *NVT* simulation with SHAKE, 1 ns *NPT* equilibration with SHAKE, and finally 10 ns *NPT* production with SHAKE. In all simulations, the temperature was set to 300 K and, in the latter two, a pressure of 1 atm was kept constant with the Berendsen algorithm.^50^ Radial distribution functions (*g*(*r*)) and root-mean square deviations were calculated with the CPPTRAJ program.^51^

Equilibrated solvent boxes were obtained by a similar procedure, involving minimization, 10 ps, and 1 ns *NVT* MD simulation without and with SHAKE, respectively, and a final 100 ns *NPT* simulation using the Berendsen barostat. Five solvent boxes were equilibrated: pure ACN, pure DCM, pure DMA, a 1:10 DMA:ACN mixture ({DMA;ACN}), and a 1:10 DMA:DCM mixture ({DMA;DCM}).

Investigations of CE yields were performed by first simulating 50 different starting structures of solvated systems before photoinduced electron transfer for 10 ns each. The Fe­(III*)­NHC^+^ excited state of the photosensitizer was for simplicity modeled with the same force field parameters as the electronic ground state (i.e., involving one molecule each of Fe­(III)­NHC^+^, DMA, and PF_6_^–^) motivated by the fast charge-separation occurring on this system and the minimal geometrical and electrostatic differences between the ground and excited states of the Fe­(III)­NHC^+^ photosensitizer (details in Table SI.2 and Figure SI.2 in the Supporting Information). The MD simulations were run according to a previously described equilibration procedure. MD simulations of Fe­(III)­NHC^+^ and DMA show preassociation at minimum distances of ∼3 Å (defined as the minimum distance between any pair of atoms in the two molecules), see Figure SI.9. Therefore, a set of 50 frames with a ∼3 Å distance between Fe­(III)­NHC^+^ and DMA was collected from the final 50 production runs. Subsequently, 50 production runs of 1 ns were performed, changing the models to the charge-separated species instead (i.e., Fe­(II)­NHC, DMA^•+^, and PF_6_^–^). The Fe­(II)­NHC and DMA^•+^ distances were tracked during the simulation and a threshold of 6 Å minimum distance was used to define a successful CE. This cutoff distance is equivalent to the width of the first solvation sphere around Fe­(II)­NHC (Figure SI.5c,d). Charge-recombination was also included in the model by assuming deactivation of the charge-separated state if the neutral–cation pair remained in close contact for longer times than a preset charge-recombination time constant.

The DMA^•+^ dissociation free energy from Fe­(II)­NHC was also investigated using umbrella sampling.^52^ The reaction coordinate was defined as the distance between the Fe ion in Fe­(II)­NHC and the N atom in DMA^•+^, and this distance was restrained using a harmonic potential. 150 MD simulations were run for 10 ns with distance increments of 0.1 Å starting from 5 to 30 Å (ensuring good sampling overlap between windows) and a force constant of 10 kcal mol^–1^ Å^–2^. The potential of mean force (PMF) was constructed using the weighted histogram analysis method (WHAM).^53^ The standard errors (standard deviation from the mean value) were estimated at each internal coordinate using five different data sets.

### Quantum Chemical Calculations

DMA^•+^, [(DMA)_2_]^•+^, and [(DMA)_3_]^•+^ as well as the corresponding ion-pair structures interacting with a PF_6_^–^ counterion species were optimized with the B3LYP^40^ density functional theory (DFT) method with modified HF exchange (15%; B3LYP*)^41^ and the 6-311G­(d) basis set, including Grimme’s D3 empirical dispersion correction.^54,55^ The calculations were sped up by the resolution of identity (RI) approximation, RIJCOSX, using auxiliary basis set def2/J. Redox potentials were calculated relative to ferrocene (Fc­(+1/0)), and the final energies were corrected as suggested by a benchmark study of the donor and iron photosensitizers with B3LYP*.^56^ Basis set superposition error (BSSE) was accounted for by the counterpoise method for systems considering the effect of counterions on the calculated redox potentials. Binding free energy calculations were also calculated as the Gibbs free energy difference between the single monomers and the dimer or trimer structures (with and without the counterion). These calculations were performed in the ORCA 4.2.1 software.^57^

### Quantum Mechanics/Molecular Mechanics

To assess the influence of radical-cation donor dimerization ([(DMA)_2_]^•+^) on CE, the delocalization of the electron density between the two monomers was studied by conducting QM/MM MD simulations, including one [(DMA)_2_]^•+^ dimer in the QM region. The initial dimer structures were obtained by applying a force constant constraint between DMA^•+^ and one DMA during MM equilibration. The calculations were performed with the AMBER interface to the ORCA program,^58^ utilizing the semiempirical PM3 method.^59,60^ Since periodic boundary conditions are not available with this approach, a larger solvation box with a solute-to-edge distance of 40 Å was considered, and a cutoff of 45 Å (full box) was used for the electrostatic embedding (only the QM region is polarized by the solvent). The time step was reduced to 0.5 fs. Umbrella sampling was also conducted in the QM/MM scheme but with a reduced sampling range to reduce the computational cost. We considered only Fe–N distances between 8 Å and 15 Å (sufficient for describing diffusion through the first and second solvation shells) with a spacing of 0.2 Å (and a force constant of 10 kcal mol^–1^ Å^–2^). We sampled 100 ps per window, after 50 ps of QM/MM equilibration in the *NPT* ensemble (previously equilibrated at the MM level), which in total comprises a collection of 3.6 ns of QM/MM sampling in each solvent.

The dimerization and hole-hopping were further investigated by DFT in a QM/MM MD scheme. The QM part, comprising the [(DMA)_2_]^•+^ dimer and the counterion PF_6_^–^, was described by the B3LYP* functional and the 6-31G­(d) basis set, incorporating D3 dispersion. The MM part involved the photosensitizer and the DCM solvent. The simulations were performed for six different starting structures, in which the counterion is interacting with one of the monomers, to assess the counterion effect on charge localization. The system was equilibrated for 5 ps, after a previous equilibration of 30 ps with the semiempirical method PM3, and a final sampling of 10 ps. After the 10 ps sampling, an additional DMA molecule was moved from the MM region to the QM region for three of the QM/MM trajectories. The system was equilibrated again for 5 ps, and an additional 5 ps of production was run.

## Results and Discussion

### Solvent Response to Photoinduced Electron Transfer

First, solvent orientations around isolated DMA and FeNHC molecules were compared before and after photoinduced electron transfer in the {DMA;ACN} and {DMA;DCM} solvent mixtures with 1:10 solvent ratios (∼1 M DMA). According to the g­(r)­s, the first solvation shell around the Fe­(III)­NHC^+^ appears at ∼7 Å, in agreement with previous observations,^39^ and the structure of the solvent remains relatively unchanged after the charge-separation process (see Figure SI.5). In contrast, there is a significant solvent response in the vicinity of DMA when it is promoted by electron oxidation to DMA^•+^, as can be seen in Figure 2a–d. This difference in solvent response reflects a more efficient charge redistribution and internal screening by the extended molecular structure of the FeNHC complex compared to the smaller DMA molecule.

**Figure 2:**
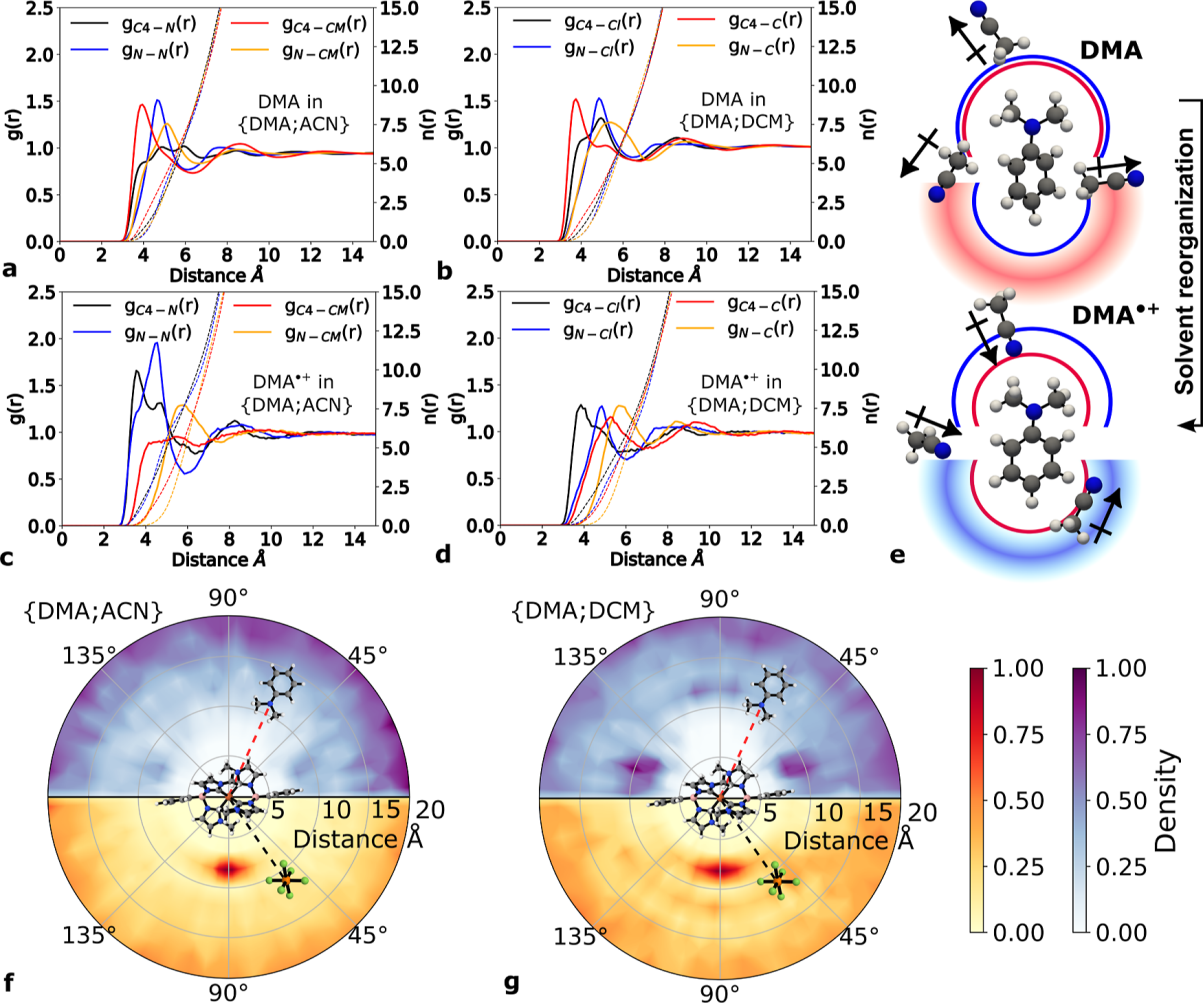
(a–d) Radial distribution functions (*g*(*r*)) of DMA and DMA^•+^ in the {DMA;ACN} and {DMA;DCM} solvents. The full lines correspond to the *g*_C4–X_(*r*) and *g*_N–X_(*r*) functions for the DMA C4 or N atoms (atom numbers shown in Figure 1), the ACN atoms X = N or CM (C in CH_3_), and the DCM atoms X = Cl or C. The dashed lines are the corresponding integrated functions (*n*(*r*)). (e) Snapshots representing the solvent orientation around the DMA and DMA^•+^ amine group and benzene ring. Curved lines schematically represent CH_3_(ACN) (in blue) and N­(ACN) (in red) distributions around N­(DMA) and C4­(DMA). Diffuse-shaded curved regions illustrate more movement flexibility. (f,g) Angular and radial distribution of DMA^•+^ (top blue hemisphere) and PF_6_^–^ (bottom orange hemisphere) with respect to Fe­(II)­NHC in {DMA;ACN} and {DMA;DCM}. The considered distances and angles are X–Fe­(Fe­(II)­NHC) and B­(Fe­(II)­NHC)–Fe­(Fe­(II)­NHC)–X, where X is either N­(DMA^•+^) or P­(PF_6_^–^).

An analysis of the first solvation shell surrounding neutral DMA reveals a structured solvent distribution, characterized by sharp and directional peaks in the *g*(*r*)­s, regardless of the solvent type. About seven solvent molecules are accommodated in this first solvation shell of ∼3 Å width. The solvent is particularly ordered around the DMA aromatic ring, as suggested by the *g*_C4–X_(*r*) (X = CM or C) peaks at a distance of 4 Å, where the electropositive group of the solvent (CH_3_ in ACN or CH_2_ in DCM) is pointing toward the C4 atom in DMA (atom numbering in Figure 1). On the other hand, there is no distinct solvent arrangement in the vicinity of the amine group, as evidenced by the *g*_N–X_(*r*) (X = N, CM, Cl, or C), all with peaks localized at similar distances, ∼5 Å. Schematic representations of the solvent orientations are shown in Figure 2e.

The change in the charge distribution caused by the oxidation of DMA to DMA^•+^ induces a drastic reorientation of the solvent around the aromatic molecule. The peaks at 3.7 Å and 3.8 Å found in *g*_C4–N_(*r*) and *g*_C4–Cl_(*r*), respectively, suggest that the solvent molecules around DMA^•+^ flip completely (the electronegative groups of the solvent are pointing toward DMA^•+^) compared to their initial orientations around the neutral DMA molecule. Therefore, the ^2^LMCT quenching by DMA causes significant solvent reorganization, in particular, around the phenyl ring of the DMA donor, as a consequence of the extra charge distributed around this ring. The more prominent intensity and sharpness of the solvent peaks found for ACN show that the solvent organization near DMA^•+^ is more pronounced in ACN than in the less polar DCM solvent. It is worth noting that the influence of other intermediates in the excitation of Fe­(III)­NHC^+^ was neglected since the ^2^LMCT state quenching is highly efficient at high DMA concentrations (quenching rate of 1.25 ps^–1^).^26^

This analysis of the solvent structure and reorganization during the bimolecular electron transfer process highlights the importance of the molecular size and its capability to localize or delocalize the charge beyond considerations of the solvent polarity and the net charge of the donor–acceptor pair. The subtle differences in the solvent reorganization between ACN and DCM cannot by themselves explain the observed difference in CE yields. This prompted us to conduct further studies of the system dynamics, as described in the following sections.

An analysis of the distribution of DMA^•+^ around Fe­(II)­NHC, shown in Figure 2f,g (upper blue hemisphere), reveals a tendency for DMA^•+^ to migrate away from the reduced (uncharged) photosensitizer (see also Figure SI.9). However, there is a subtle difference in the distributions between the two solvents at short Fe­(II)­NHC–DMA^•+^ distances. Figure 2g ({DMA;DCM} solvent) shows a preferential position of DMA^•+^ around 10 Å from the photosensitizer, which matches with the structural pockets arising between the scorpionate ligand and the metal, but the corresponding peaks are much weaker in the {DMA;ACN} simulations. A similar effect has been observed for the counterion (lower red hemisphere in Figure 2f,g), although the distributions show the preferred placement near the central region around the photosensitizer. These results clearly indicate stronger Fe­(II)­NHC–DMA^•+^ (neutral–cation) and Fe­(II)­NHC–PF_6_^–^ (neutral–anion) pairing in the less polar {DMA;DCM} and pure DCM solvents (Figure SI.8).

### Radical-Cation Cage-Escape Dynamics

Next, the driving force toward DMA^•+^ solvation after charge-separation was investigated by classical MD simulations in both {DMA;ACN} and {DMA;DCM} solvent mixtures. In order to account for the solvent reorganization energy caused by the electron transfer, we started by equilibrating the ground-state system, i.e., before charge-separation. We then selected 50 equilibrated snapshots where Fe­(III)­NHC^+^ and DMA are in close contact (ca. 3 Å), changed the charges of the two molecules to match the charge-separated state, and ran MD simulations during which the dissociation of DMA^•+^ from the Fe­(II)­NHC PS was tracked. Figure 3a,b shows the time evolution of the minimal Fe­(II)­NHC–DMA^•+^ distance as well as the calculated CE yields in both solvents (detailed protocol in the Computational Details section). As charge-recombination of the system in dichloromethane is unknown, we have assumed three scenarios: (i) no charge-recombination, (ii) 50 ps charge-recombination, and (iii) 5 ps charge-recombination.

**Figure 3:**
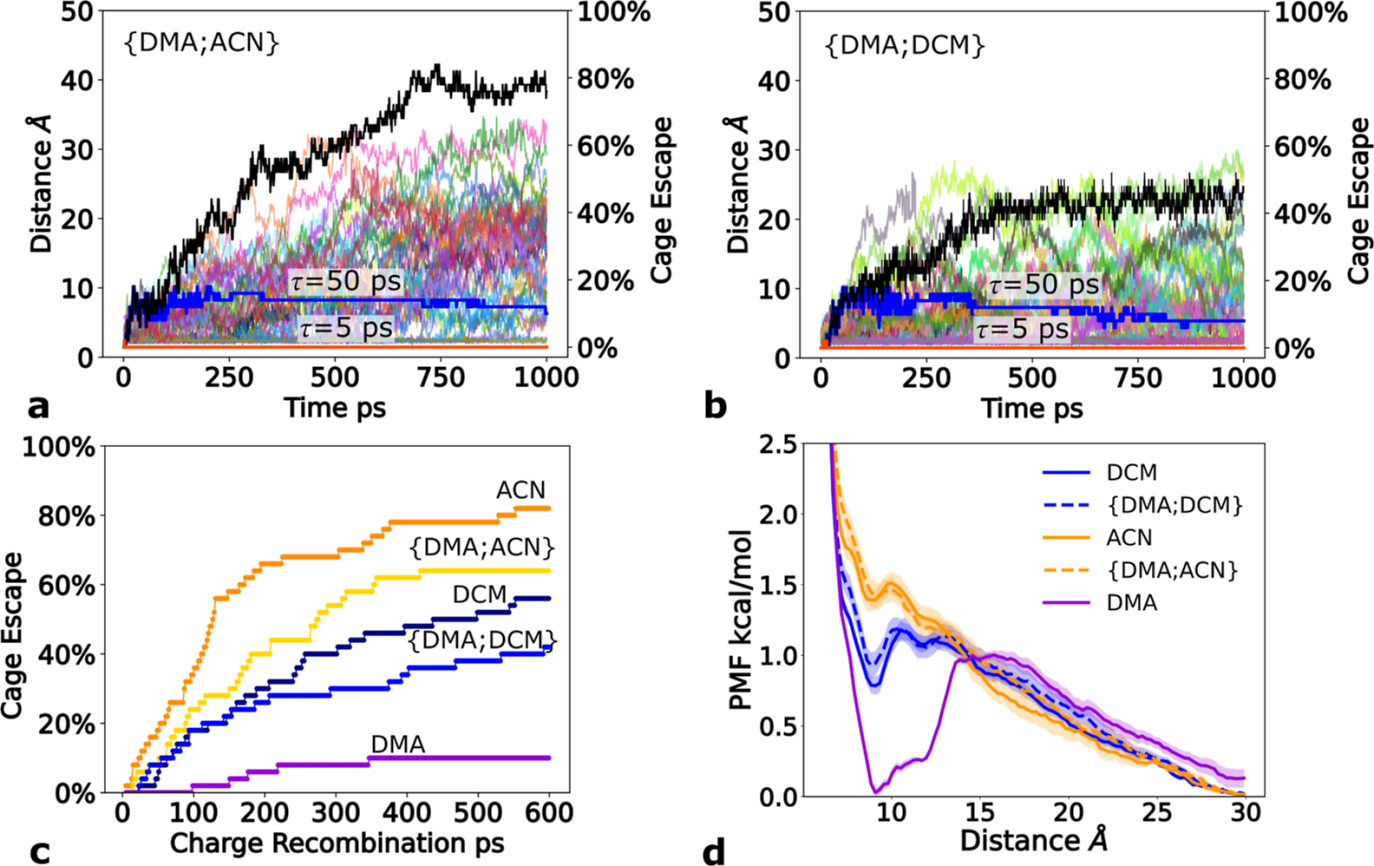
Minimal Fe­(II)­NHC–DMA^•+^ distances (in Å) as a function of time for 50 trajectories (thin color lines) in (a) {DMA;ACN} and (b) {DMA;DCM} solvent mixtures. Calculated cage-escape yields are displayed in black neglecting charge-recombination, in blue with 50 ps charge-recombination time constant, and in orange with 5 ps charge-recombination time constant. (c) Cage-escape yields achieved after 1 ns of sampling for a range of charge-recombination time constants in ps. (d) Potential of mean force (PMF) for the DMA^•+^ solvation along the Fe­(Fe­(II)­NHC)–N­(DMA^•+^) distance for five different solvents. The shaded region illustrates the standard error.

The negligible CE yields achieved under the assumption of 5 ps charge-recombination time constant in both solvents (matching the results from transient absorption spectroscopy measurements in ACN)^26^ reflect the poor competitiveness of diffusion compared to electron back-transfer. Even when the charge-recombination time constant is increased to 50 ps, the resulting CE yields of 10% and 8% for {DMA;ACN} and {DMA;DCM}, respectively, are still low when compared to the experimental findings. Notable CE yields in DCM were observed only at long recombination times (Figure 3c). In the idealized case where charge-recombination is disregarded, significantly larger CE yields were observed in {DMA;ACN} (∼80%) than in {DMA;DCM} (∼45%). This is clearly the reverse trend compared to what was observed experimentally (∼2% in ACN and ∼60% in DCM) by comparative actinometry measurements.^27^ Therefore, to support the 30 times larger CE yields in DCM, the recombination dynamics would need to be decelerated by a factor of ∼100. These findings suggest that other factors must modify the solvent interplay beyond the regular solvation propensity of the product species.

Experimental work has shown a strong correlation between the concentration of the donor and the presence of photoproduct for DMA concentrations >200 mM.^26,27^ Therefore, we also investigated the CE response in pure ACN, DCM, and DMA solvents. These results (reported in Figures SI.14 and 15) show CE yields of ∼2% in pure ACN, ∼0% in pure DCM, and ∼0% in pure DMA for 5 ps recombination times and ∼20% in pure ACN, ∼5% in pure DCM, and ∼0% in pure DMA for 50 ps recombination times. As the donor concentration increases, the solvation of DMA^•+^ decreases, so that the escape success is almost negligible in pure DMA. Despite the general increase of the diffusion yields in pure ACN and DCM, DMA^•+^ solvation is still favored in ACN over DCM, in agreement with the simulations in the {DMA;ACN} and {DMA;DCM} mixtures. The stronger directional solvent reorganization in ACN (shown in Figure 2) around the positive charge of DMA^•+^ also agrees with the stronger driving forces toward solvation.

Further investigations of the free energy of the cage escape of DMA^•+^ were performed by umbrella sampling. Figure 3d illustrates the PMF profiles in the five solvents. The overall tendency is toward complete DMA^•+^ solvation as indicated by the downhill trend of the PMF with respect to the Fe­(Fe­(II)­NHC)–N­(DMA^•+^) distance. The PMFs of the two studied ACN solvents show a weak local minimum (∼0.2 kcal/mol at 9 Å) for the bound state and therefore indicate that the dissociation is almost barrierless. In contrast, there are slightly more pronounced bound local minima at 9 Å in the two DCM solvents and in pure DMA, with activation barriers of ∼0.4 and 1.1 kcal/mol, respectively. Despite the activation barrier being twice as high in DCM as in ACN, in agreement with the observed CE trends, the shallow potential in both cases suggests that the CE dynamics is essentially diffusion limited. Furthermore, the similar energy barriers found for the solvation of neutral organic bimolecular systems in both acetonitrile and dichloromethane agree with a weak pairing in the studied radical-cation–neutral system.^61^

These findings suggest that the CE yield of DMA^•+^ is significantly affected by the DMA concentration, reducing the DMA^•+^ mobility at high DMA concentrations. Thus, a high DMA concentration would favor charge-recombination (because the photosensitizer and donor form a complex for a longer time), but it would also favor the charge-separation process by the higher concentration of available donor molecules in the vicinity of Fe­(III)­NHC^+^. This implies that a balance is needed for the DMA concentration to maximize the efficiency of charge-separation and minimize charge-recombination.

### Ion-Pairing and Hole-Hopping Cage Escape

Although the experiments showed lower CE yields in more polar solvents (5% in ACN) for the Fe­(II)­NHC–DMA^•+^ system, the range of CE yields in various amine systems is very broad (2–100%) even for the same solvent.^11,21,25,30,62–65^ This prompted us to study several alternative CE mechanisms.

Features in the near-infrared spectrum of amine derivatives and similar organic moieties have been attributed to the formation of dimers or radical-cation dimers.^66–73^ We explored the dimerization of radical-cation DMA^•+^ and a neutral DMA molecule. Optimized geometries of the dimer obtained at the B3LYP*-D3/6-311G­(d) level are depicted in Figure 4a. We explored a sandwich-stacking mode by *N*–π ([(DMA)_2_]_I_^+^) or *N–N* stacking ([(DMA)_2_]_II_^+^) similar to what has been observed in parent aromatic cation-radical dimers.^74,75^ Analysis of spin density shows that the electron hole is equally distributed between the two monomers (50% of the spin population on each monomer; cf. shown in Figure 4a). This study was further extended to the stacking of one DMA^•+^ and two DMA molecules, yielding trimers. A similar behavior of the spin density was also seen for the trimers, although the hole is preferably localized on the middle monomer (∼50%, compared to ∼25% for each of the side monomers).

**Figure 4:**
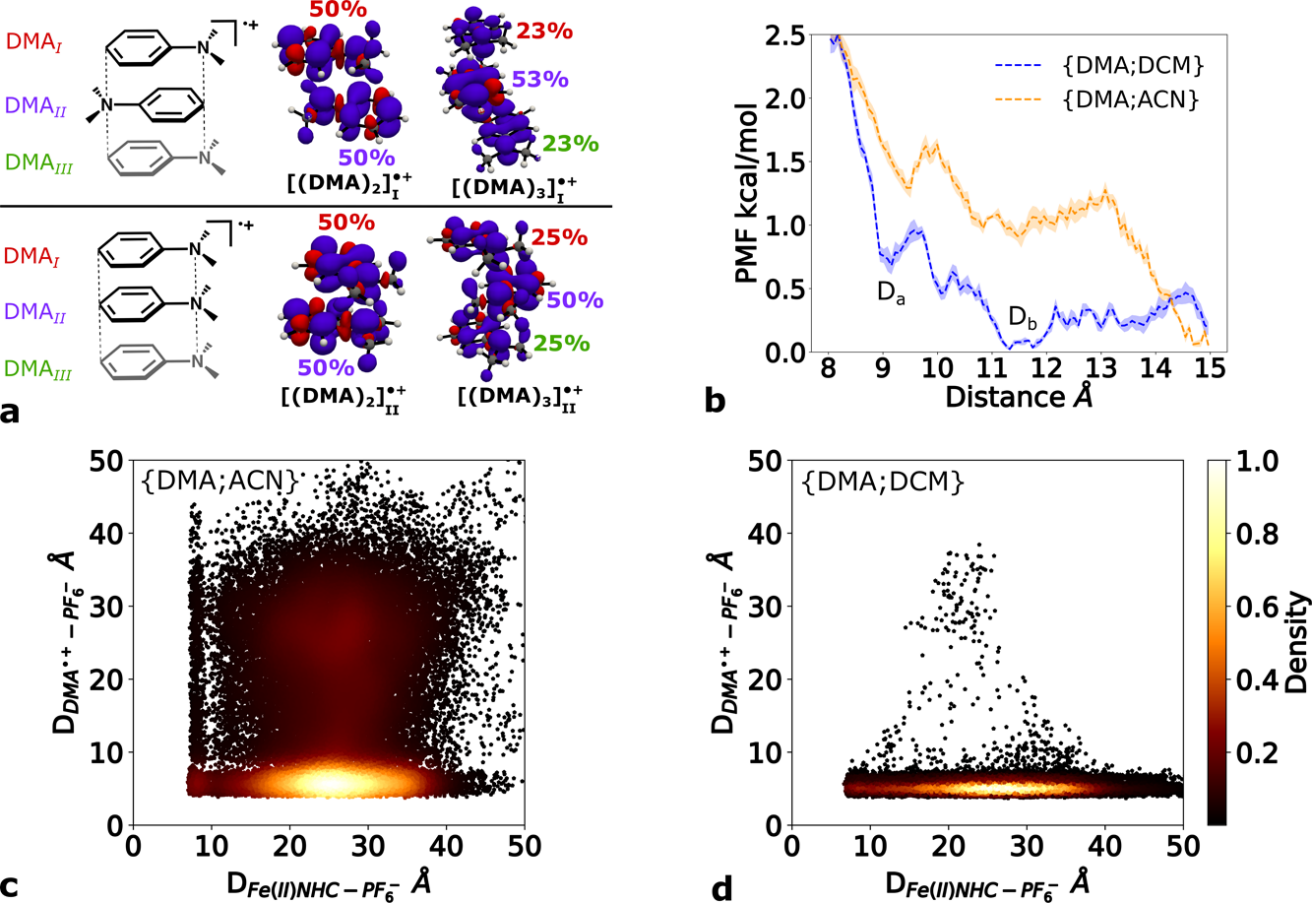
(a) Optimized dimer and trimer structures in two stacking modes, ([(DMA)_*n*_]_I_^•+^) and ([(DMA)_n_]_II_^•+^) (*n* = 2, 3), together with the spin density and the relative spin population of each monomer. (b) Potential of mean force (PMF) for the [(DMA)_2_]^•+^ dissociation from Fe­(II)­NHC in the {DMA;ACN} and {DMA;DCM} solvent boxes. The shaded region illustrates the standard error. Fe­(Fe­(II)­NHC)–P­(PF_6_^–^) and N­(DMA^•+^)–P­(PF_6_^–^) distances for a collection of snapshots from 500 ns MD simulations of the charge-separated state in (c) {DMA;ACN} or (d) {DMA;DCM}. The color code is based on the normalized density (darker for low data density and lighter for high data density).

The close contact between DMA^•+^ and DMA molecules may alter the propensity for dimer diffusion, particularly for less polar environments due to better charge redistribution. Although the calculated potentials for a single DMA molecule revealed low activation barriers, small changes on the potential can have an effect on the early dynamics, particularly for the current system with charge-recombination on the order of few picoseconds. Hence, we performed a QM/MM MD study of the dissociation of the dimer from Fe­(II)­NHC, including the dimer in the QM region to improve the description of the polarization of the monomers. We used umbrella sampling to estimate the CE efficiency in {DMA;ACN} and {DMA;DCM} solvents. The resulting PMFs in Figure 4b show a profile with two discrete minima for both solvent mixtures, one localized at ∼9 Å (*D*_a_) and a second one at ∼13 Å (*D*_b_; both minima correlate with those found in Figure 3d). The first minimum, corresponding to the first solvation shell, shows that the energy barrier for radical-cation dimer dissociation in a {DMA;DCM} environment is 0.3 kcal/mol lower than that for the radical-cation monomer. In contrast, the PMF profile in {DMA;ACN} solution changes from being barrierless to an activated process with a barrier of 0.3 kcal/mol. These findings support the notion that the polarity of the solvent could enhance the dimer CE yield in the early stage of the charge-separated state. However, the differences between the two solvents are too small to readily support the larger CE yield in DCM.

It has previously been observed for the charge-separated state of [Ru­(II)­(bpy)_3_]^2+^ and MV^2+^ that the CE can be influenced by the formation of pairs between the reactants and the electrolyte ions and that this effect can be tuned by changing the ions.^76,77^ Therefore, ion-pairing between DMA^•+^ and the counterion PF_6_^–^ may affect the CE yields and relate to the capability of the solvent to break such interactions. An in-depth analysis of cation–anion distance distributions in 50 independent 10 ns MM MD simulations of the charge-separated state reveals distinct ion-pair distances depending on the solvent ({DMA;ACN} or {DMA;DCM}). Figure 4c indicates two preferred conformations for the {DMA;ACN} simulations, both involving Fe­(II)­NHC dissociated from DMA^•+^, viz., either a DMA^•+^–PF_6_^–^ ionic pair or DMA^•+^ dissociated also from PF_6_^–^. On the other hand, the corresponding plot for the {DMA;DCM} solvent shows that the DMA^•+^–PF_6_^–^ ionic pair conformation strongly dominates. This distinct cation–anion-pairing dominates before electron transfer, where the charged photosensitizer and the counterion (Fe­(III)­NHC^+^–PF_6_^‑^) are strongly coupled (see Figure SI.13). Therefore, the Fe­(II)­NHC and PF_6_^–^ species will also be in the vicinity of each other immediately after charge-separation in DCM solvents. Thus, the polarity of the solvent plays a key role in the balance between the formation of either cation–anion pairs or solvated ions. This difference in the behavior of the charge-separated species and the counterion between the solvents prompted two considerations: the potential hindering of the charge-recombination process by the neutralization of the radical-cation charge-separated species and the possibility that the counterion leads to the CE dynamics.

With the aim of evaluating the effect of dimerization and trimerization on the driving forces for electron transfer steps, redox potentials were estimated from DFT calculations for DMA^•+^ in monomer and two potential dimer and trimer stacking styles (see Figure 5a,b; the exact values are listed in Table SI.3). The calculations demonstrate that aggregation shifts the redox potentials to more negative values compared to the monomer, i.e., to a stronger reductive power. For example, the redox potential of [(DMA)_3_]_II_(+1/0) is reduced by ∼0.60 V compared to DMA in both ACN and DCM. Naturally, such large changes in the redox potential (0.3–0.6 V) by aggregation can strongly affect the charge-recombination efficiency. To further assess the impact of the counterion, redox potentials were recalculated, including also the counterion in the DFT calculations. Interestingly, all reduction potentials involving the counterion are shifted to even more negative values, by ∼0.1 and ∼0.3 V for ACN and DCM, respectively. Therefore, subtle changes in the ion-pairing may significantly affect the driving forces toward charge-recombination.

**Figure 5:**
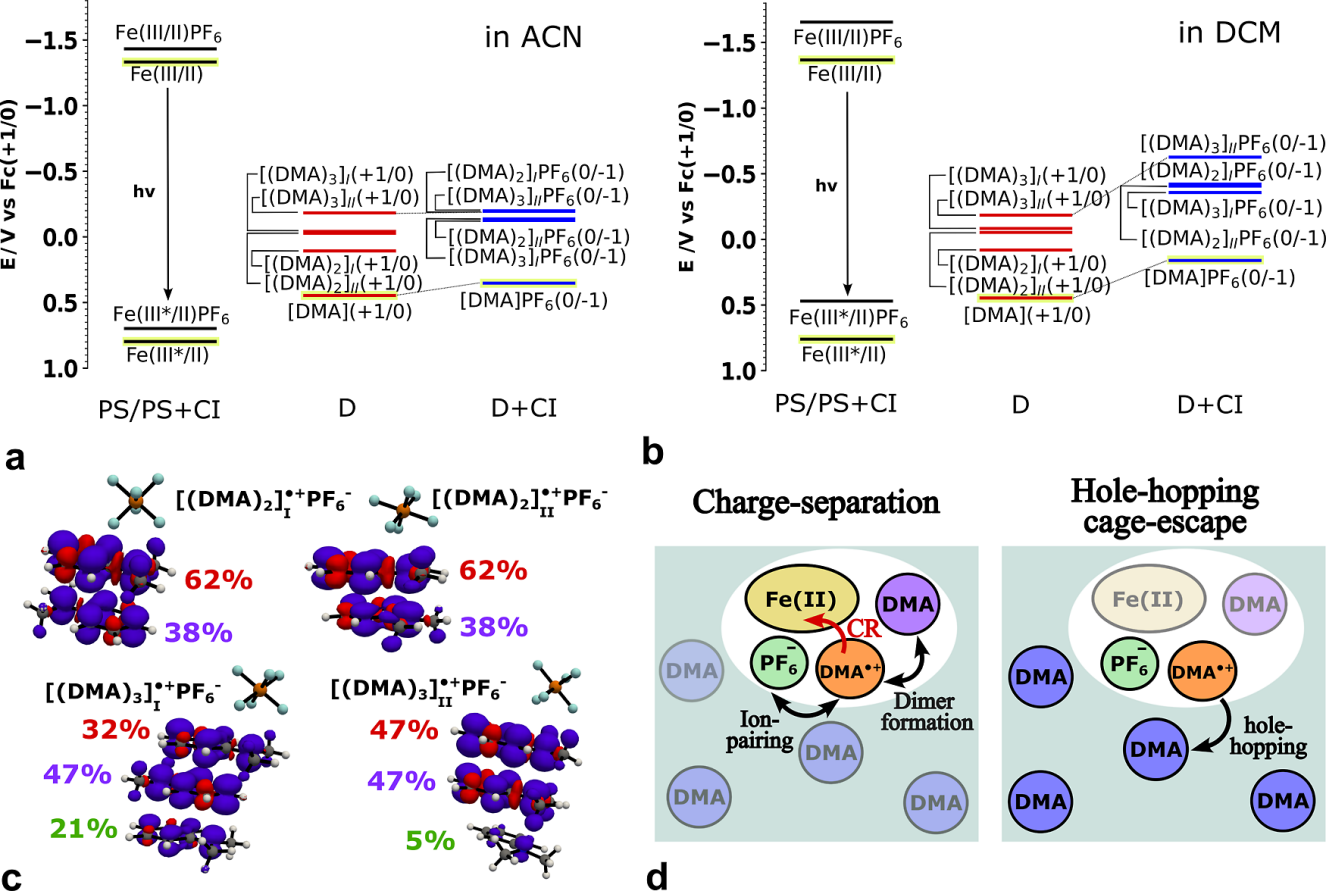
Calculated reduction potentials of the photosensitizer (PS) Fe­(III/II)­NHC and electron donor (D) DMA­(+1/0), [(DMA)_*n*_]_I_(+1/0), and [(DMA)_*n*_]_II_(+1/0) (*n* = 2, 3) without (red) and with (blue) the counterion (CI) in (a) acetonitrile (ACN) and (b) dichloromethane (DCM). The Fe­(III*/II) photoreduction potential was calculated using the experimental excited-state energy gap.^25^ All reduction potentials are expressed with respect to the ferrocene redox couple (Fc­(+1/0)). (c) Optimized dimer and trimer structures with the presence of the PF_6_^–^ counterion together with the spin density representation and the relative spin population of each monomer. (d) Schematic representation of the hole-hopping cage-escape mechanism, ion-pairing, and dimer formation for the studied system. Charge-recombination (CR) is also highlighted in red.

The phenomenon of transferring a positive charge between similar chemical units, known as hole-hopping, has been suggested as a mechanism that can contribute to the overall charge transfer/migration in some cases, for example, between residues in proteins and for Ru­(II) photosensitizers attached to electron conductive films.^78,79^ The tendency of DMA^•+^ to aggregate due to significant neutral–radical-cation stabilization noted above encouraged us to also consider the possibility that hole-hopping contributes to the CE of DMA^•+^.^80,81^ QM calculations of the dimer and trimer systems with explicit counterions show a redistribution of the spin density population toward the monomer closest to the counterion by ∼10%, as shown in Figure 5c.

We further conducted six QM/MM MD simulations involving one [(DMA)_2_]^•+^ dimer and one PF_6_^–^ counterion at the QM level in a classical DCM solvent to further characterize the ion-pairing dynamics and associated electronic signatures. The spin densities of each monomer and the counterion were tracked over a 10 ps trajectory to assess the hole location over time. The results from two representative simulations are shown in Figure 6a,b (the four remaining simulations are included in Figure SI.17). Three of the simulations showed that the hole is delocalized rather evenly between the two monomers during the simulation, as illustrated in Figure 6a. On the other hand, the other three simulations showed that the hole is essentially localized on one monomer throughout the simulation, as illustrated in Figure 6b. The hole localization, which occurred after ∼3 ps, took place without any significant change in the monomer–monomer distance (see all distances in Figure SI.19), which was rather constant at ∼2.2 Å, suggesting that solvent reorganization may play a key role for this process. The two different hole dynamics observed in the two simulations suggest that there can be a competition between the formation of [(DMA)_2_]^•+^ or DMA^•+^–PF_6_^–^ pairs that is likely to be sensitive to experimental conditions such as electron donor concentration. An in-depth analysis of the trajectories (Figure 6c,d) indicates a moderate tendency for the counterion to migrate if the DMA_II_–DMA_I_ coupling is initially more favored than the charge neutralization and another DMA molecule is in the vicinity in the MM region.

**Figure 6:**
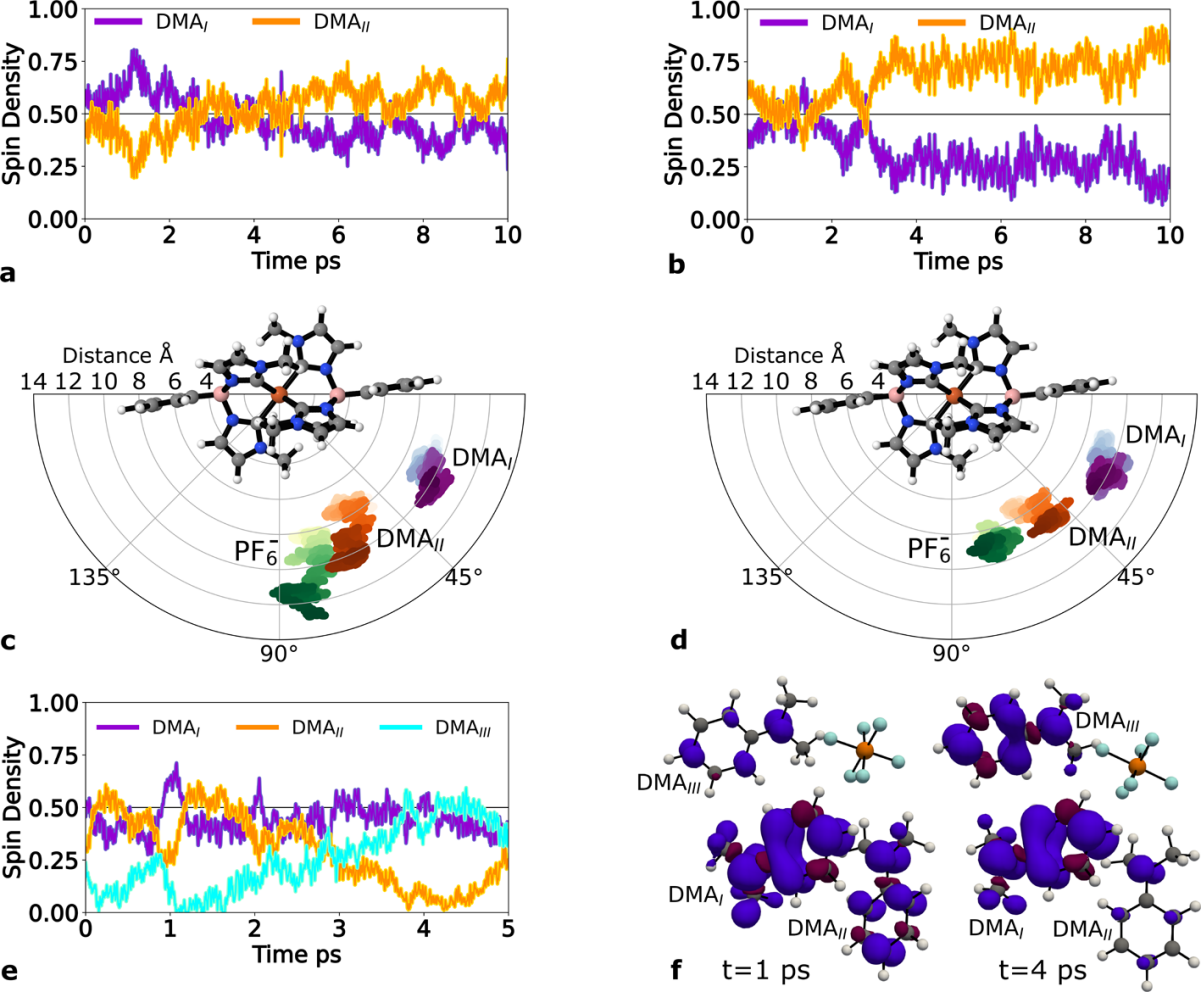
(a,b) Spin density of the radical-cation dimer (the spin on each monomer is shown in violet and orange, respectively) during 10 ps of two QM/MM MD simulations. (c,d) Angular and radial trajectories of monomer DMA_I_, monomer DMA_II_, and PF_6_^–^ with respect to Fe­(II)­NHC during the 10 ps QM/MM MD simulations. The distances and angles are calculated for the Fe­(Fe­(II)­NHC), N­(DMA_I_), N­(DMA_II_), and P­(PF_6_^–^) atoms. The color gradients illustrate the time progression (light in the start and dark at the end). (e) Spin density evolution of the radical-cation trimer (the spin on the three monomers is shown in violet, orange, and blue, respectively) during 5 ps of a QM/MM MD simulation. The corresponding spin densities at 1 and 4 ps are shown in (f).

In the three systems, the QM/MM MD simulation was continued by including a third neighboring DMA molecule in the QM region. Interestingly, two of the simulations show a hole transfer from one monomer in the initial dimer to the third incorporated DMA monomer (DMA_III_), which interacts with the counterion. Figure 6e is a representative case of hole-hopping occurring in only 3 ps, a time range that can compete with the charge-recombination. Future work modeling the charge-recombination by ab initio MD could provide a solid basis for comparison with the calculated hole-hopping. We also observed that the monomer aggregation dynamics preferably involves the formation of dimers, and the hole migration is aided by the formation/depletion of a dimer and a neighboring monomer (see Figure 6f). The calculations of the dimer and trimer binding free energies also support the favorable driving forces toward dimerization, which is more preferred in DCM than in ACN (all energies are listed in Table SI.6). Furthermore, the formation of radical-cation-dimer–counterion pairs was found to be favorable only in DCM (−1.38 kcal/mol). Thus, hole-hopping is a possible competing cage-escape mechanism for DMA^•+^ diffusion, facilitated by ion-pairing interactions.

## Conclusions

This work expands our knowledge of solvent cage-escape mechanisms after charge-separation involving the [Fe­(III)­(phtmeimb)_2_]^+1^ photosensitizer and the dimethylaniline donor molecule through a combination of classical molecular dynamics simulations and quantum chemical calculations.

We have addressed recent experimental observations of charge transfer rates and cage-escape-mediated photoredox product formation, including nontrivial solvent and concentration dependencies, by modeling the diffusion of the charge-separated photosensitizer and donor molecule in two solvents (ACN and DCM), as well as the impact of electron donor concentrations on the cage-escape rates. An initial comparison of cage-escape yields in the two solvents revealed almost twice as large cage-escape yields of DMA in ACN as in DCM, contrary to what was observed in the actinometry experiments. This discrepancy between the simulation and experimental evidence motivated further investigations of alternative cage-escape routes as well as other solvent responses beyond diffusion. Analysis of the solvent structure around the electron donor, before (DMA) and after (DMA^•+^) photoinduced electron transfer, showed rather similar solvent response regardless of the solvent. Therefore, we considered two further intermolecular interactions of DMA^•+^, viz., with the counterion and/or with surrounding (neutral) DMA molecules, that may be present at higher donor concentrations.

An analysis of the coupling dynamics between the PF_6_^–^ counterion and DMA^•+^ or the ground-state photosensitizer prior to the photoinduced charge-separation revealed a particularly strong tendency for ion-pairing in the less polar DCM solvent (cf. Figure 4d). A capability of DMA^•+^ to form radical-cation dimers in solution was also computationally confirmed, and these aggregates were observed to include a significant degree of hole delocalization between the interacting monomers. At high DMA concentration, where such interactions can be expected, the simulations suggest that hole-hopping between neighboring electron donors can contribute to the charge transfer/migration dynamics on fast picosecond time scales and that such dynamics can be correlated also with counterion stabilization and migration effects to yield an overall stabilization of DMA^•+^. Such faster photoproduct charge stabilization appears particularly important in cases such as the here studied one, where cage-escape yields can be suppressed by fast charge-recombination that competes with slow diffusion-driven cage-escape dynamics. Both delocalization and hopping of the (DMA^•+^) charge can contribute to the hole mobility, and counterion migration to form ion-pairing interactions also provides an additional mechanism that can contribute to suppress charge-recombination in favor of ultimate cage escape. The stabilization of DMA^•+^ by intermolecular interactions involving a combination of ion-pairing and radical-cation dimer formation can also significantly modify the redox potential of DMA compared to the monomer radical-cation that is formed initially during the photoinduced charge-separation. In particular, the simulations suggest that the driving forces toward charge-recombination are significantly reduced through competing intermolecular interactions and that this effect is particularly pronounced in low-polarity solvents such as DCM.

Despite the limitations of our simplified representation of an intricate multielectron and multichannel mechanism, our simulations demonstrate that intermolecular interactions between DMA^•+^ and other species provide additional competing factors and pathways that may be important from both a kinetic and thermodynamic perspective to stabilize the oxidized photoproduct on picosecond time scales. These factors provide important ways to favor photoproduct stabilization and migration over competing charge-recombination. Thus, they potentially play important roles in enhancing successful cage-escape dynamics. Several of the factors discussed here are expected to depend on the conditions of the investigated system, including the specific combination of photosensitizer, electron acceptor/donor, and counterion, as well as the solvent concentration and polarity. It is worth remembering that the present case is fundamentally different in terms of the intermolecular charge interactions compared with typical systems, such as Ru­(II)-driven photoredox reactions, where both the photosensitizer and the electron donor are positively charged after the charge-separation. It would be interesting in the future to consider how the intermolecular interactions discussed here contribute to cage escape in a broader range of systems with the long-term goal to reach a more comprehensive understanding of this key aspect of photoredox catalysis and to potentially suggest improved light harvesters.

## Supplementary Material





## Notes

AMBER force field files for Fe­(II)­NHC, Fe­(III)­NHC, and PF_6_^–^ molecules (ZIP)
Force field validation data, solvent cages, additional distribution functions and solvent cage-escape dynamics, and detailed analysis of QM/MM MD simulations (PDF)
