# Supplementary material for: Understanding Anomalous Cage-Escape Dynamics in Photoredox Processes Driven by a Fe(III) N‑Heterocyclic Carbene Complex
Source: J Am Chem Soc. 2025 Jul 30;147(35):31509–20. doi: 10.1021/jacs.5c04296 (PMC12412107; doi:10.1021/jacs.5c04296)
Supplement: Supplementary file 2 [file ja5c04296_si_002.pdf]

Supporting Information: 'Understanding Anomalous Cage-Escape Dynamics in Photoredox Processes Driven by a Fe(III) N-Heterocyclic Carbene Complex'

Iria Bolaño Losada[a], Ulf Ryde[a], and Petter Persson*[a]
[a]*Division of Computational Chemistry, Department of Chemistry, Lund University, Box 124, SE-22100 Lund, Sweden.*

List of Figures

SI.1 Calculated absorption spectrum of $Fe(III)NHC^+$ SI-2
SI.2 FeNHC atomic charges SI-3
SI.3 Solvent cages SI-4
SI.4 DMA and $DMA^{\bullet+}$ radial distribution functions in pure solvents SI-5
SI.5 FeNHC radial distribution functions in mixed solvents SI-6
SI.6 FeNHC-DMA radial distribution functions in pure solvent SI-7
SI.7 FeNHC-DMA radial distribution functions in mixed solvents SI-8
SI.8 Angular and radial distribution of $DMA^{\bullet+}$ and PF_6^- in pure solvents SI-9
SI.9 FeNHC-DMA distance distributions in mixed solvents SI-10
SI.10 FeNHC-PF_6^- and DMA-PF_6^- distances in mixed solvents SI-11
SI.11 FeNHC-PF_6^- and DMA-PF_6^- distances in pure solvent SI-12
SI.12 DMA-PF_6^- distances with respect to FeNHC-PF_6^- distances in pure solvent SI-13
SI.13 DMA-PF_6^- distances with respect to FeNHC-PF_6^- distances in mixed solvents SI-13
SI.14 Cage-escape yields in pure ACN and DCM SI-14
SI.15 Cage-escape yields in pure DMA SI-15
SI.16 Dimers spin density plots SI-15
SI.17 QM/MM MD electronic structure evolution in dimer systems SI-17
SI.18 QM/MM MD trajectories in dimer systems SI-18
SI.19 QM/MM MD distances over time in dimer systems SI-18
SI.20 QM/MM MD distances over time in dimer systems SI-19
SI.21 QM/MM MD electronic structure evolution in trimer systems SI-19
SI.22 QM/MM MD distances over time in trimer systems SI-20
SI.23 QM/MM MD trajectories in trimer systems SI-21

List of Tables

SI.1 Geometry details of $Fe(III)NHC^+$ SI-2
SI.2 Metal-ligand bond lengths SI-3
SI.3 Reduction potentials SI-16
SI.4 Reduction potentials with different functionals in acetonitrile SI-16
SI.5 Reduction potentials with different functionals in dicloromethane SI-16
SI.6 Binding free energies SI-17

Atoms	Joyce[a]	GbFF[a]	DFT[b]	GAFF2[b]	X-ray[c]
Fe-C1	2.040	2.055	2.031	2.024	2.008
Fe-C2	2.026	2.052	2.027	2.022	2.002
Fe-C3	1.999	2.048	2.007	2.003	1.979
Fe-B	3.215	3.421	3.215	3.274	3.202
C1-Fe-C2	85.64	82.31	85.87	87.88	86.45
C1-Fe-C3	86.67	83.36	85.55	87.87	87.00
C2-Fe-C3	87.53	84.52	86.42	87.91	87.24
Fe-B-C4	173.4	175.5	170.2	175.4	174.4

[a] Data from reference [1], [b] data from this work, [c] data from reference [2]

TABLE SI.1: Bond distances (Å) and angles (°) in the optimized Fe(III)NHC$^+$ photosensitizer at several levels of theory.

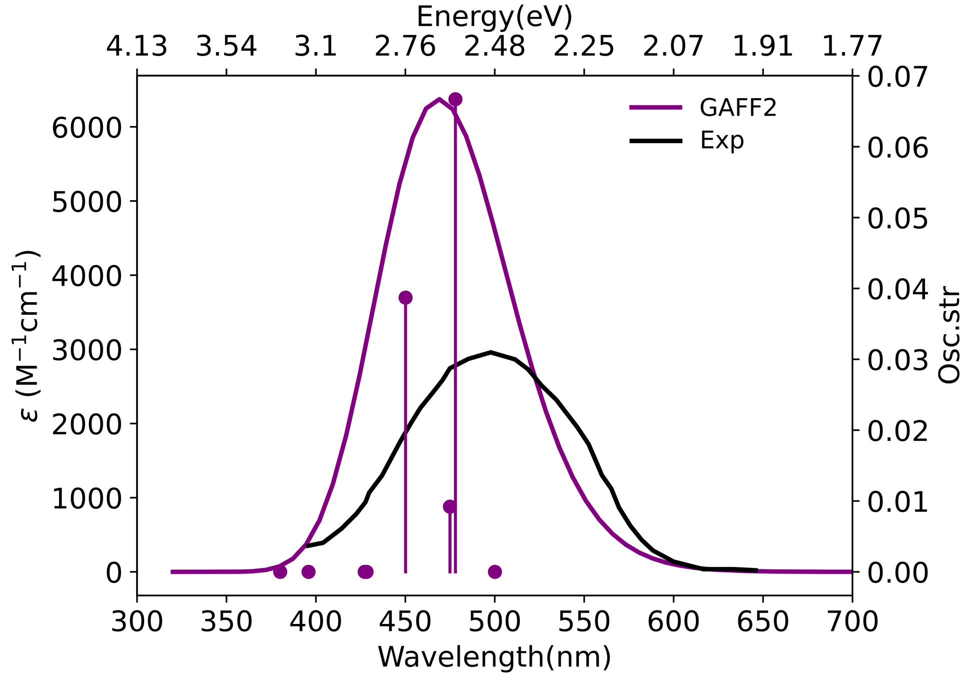

FIG. SI.1: Experimental (black) and calculated (violet) absorption spectrum with the GAFF2 optimized Fe(III)NHC$^+$ structure at the B3LYP*/6-311G(d) level of theory.

[1] Diez-Cabanes, V.; Prampolini, G.; Francés-Monerris, A.; Monari, A.; Pastore, M. Iron's wake: The performance of quantum mechanical-derived versus general-purpose force fields tested on a luminescent iron complex. *Molecules* **2020**, 25, 3084–3105.

[2] Kjær, K. S.; Kaul, N.; Prakash, O.; Chábera, P.; Rosemann, N. W.; Honarfar, A.; Gordivska, O.; Fredin, L. A.; Bergquist, K.-E.; Häggström, L.; Ericsson, T.; Lindh, L.; Yartsev, A.; Styring, S.; Huang, P.; Uhlig, J.; Bendix, J.; Strand, D.; Sundström, V.; Persson, P.; Lomoth, R.; Wärnmark, K. Luminescence and reactivity of a charge-transfer excited iron complex with nanosecond lifetime. *Science* **2019**, 363, 249–253.

Bond	Fe(III)	Fe(III*)[a]	Fe(II)[a]
Fe-C1	2.031	2.004	2.000
Fe-C2	2.027	2.002	2.000
Fe-C3	2.007	1.978	1.978

[a] Data from reference [3]

TABLE SI.2: Bond lengths (Å) in Fe(III)NHC$^+$, Fe(III*)NHC$^+$ and Fe(II)NHC optimized by B3LYP*/6-311G(d).

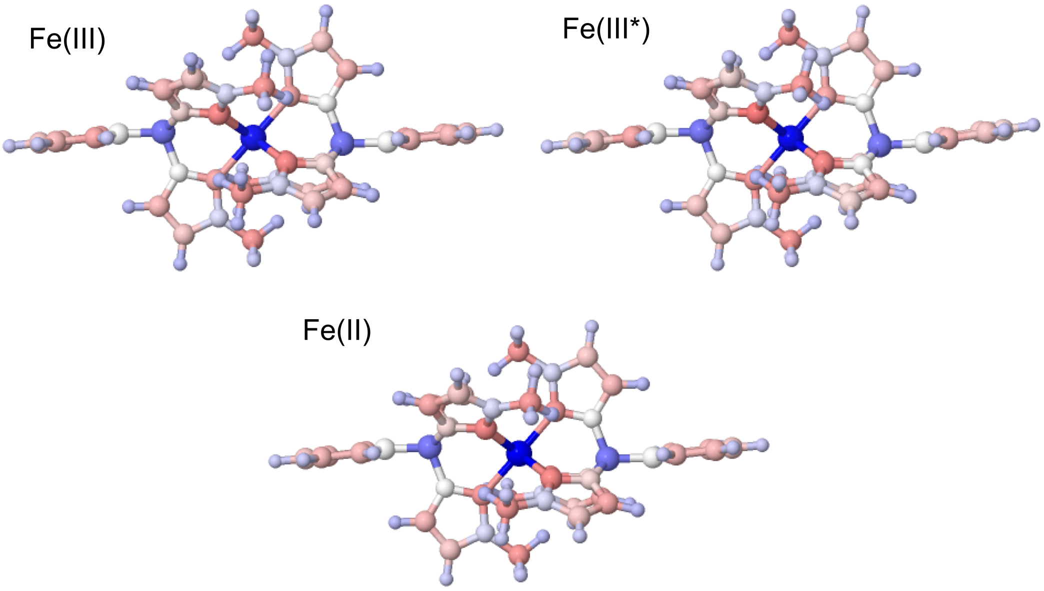

FIG. SI.2: Mulliken atomic charges for Fe(III)NHC$^+$, Fe(III*)NHC$^+$ and Fe(II)NHC. Gradient from red to blue (charges from -1 to $+1$). Data from reference [3].

[3] Rosemann, N. W.; Lindh, L.; Losada, B. L.; Kaufhold, S.; Prakash, O.; Ilic, A.; Schwarz, J.; Wärnmark, K.; Chábera, P.; Yartsev, A.; Persson, P. Competing dynamics of intramolecular deactivation and bimolecular charge transfer processes in luminescent Fe (III) N-heterocyclic carbene complexes. *Chemical Science* **2023**, 14, 3569–3579.

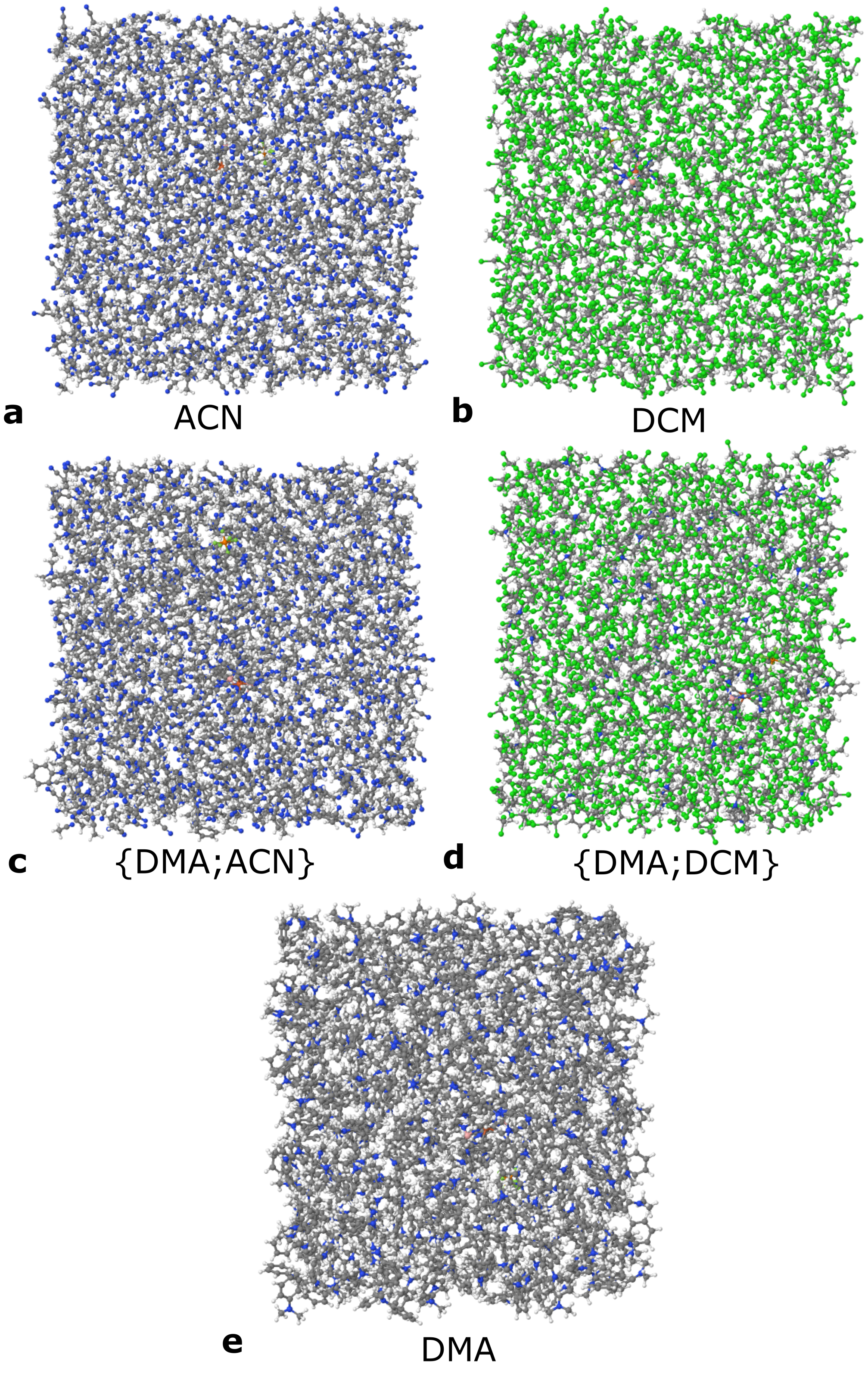

FIG. SI.3: Solvent cages used in MD simulations. (a) Pure ACN (∼ 1600 ACN molecules), (b) pure DCM (∼1300 DCM molecules), (c) {DMA;ACN} (∼100 DMA molecules; ∼1310 ACN molecules), (d) {DMA;DCM} (∼90 DMA molecules; ∼1150 DCM molecules) and (e) pure DMA (∼470 DMA molecules).

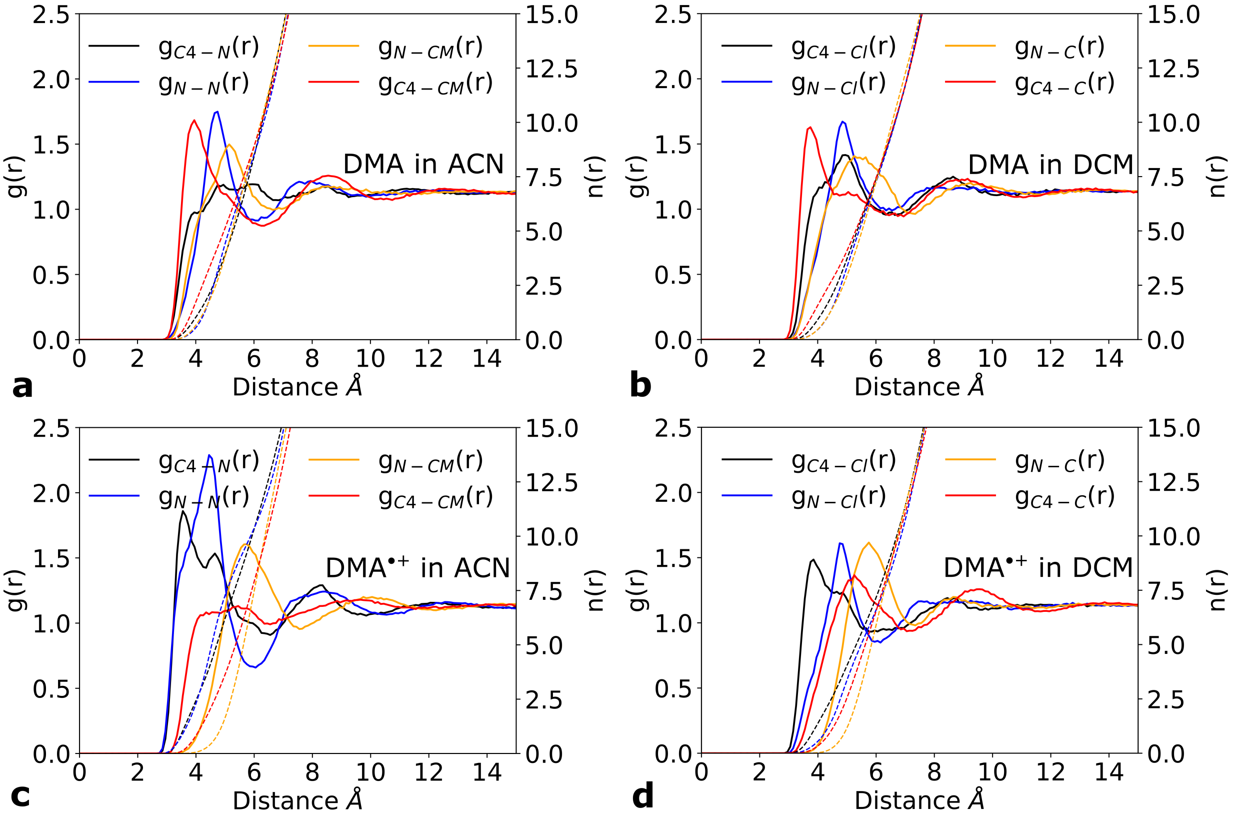

FIG. SI.4: Radial distribution functions ($g(r)$) of DMA in pure solvent (ACN and DCM), (a) DMA in pure ACN, (b) DMA in pure DCM, (c) $DMA^{\bullet+}$ in pure ACN, (d) $DMA^{\bullet+}$ in pure DCM. The bold lines correspond to the $g_{N-X}(r)$ and $g_{C4-X}(r)$ functions for the DMA N or C4 atoms, the ACN atoms X = N or CM (C in CH_3) and the DCM atoms X = Cl or C. The dashed lines are the corresponding integrated functions ($n(r)$).

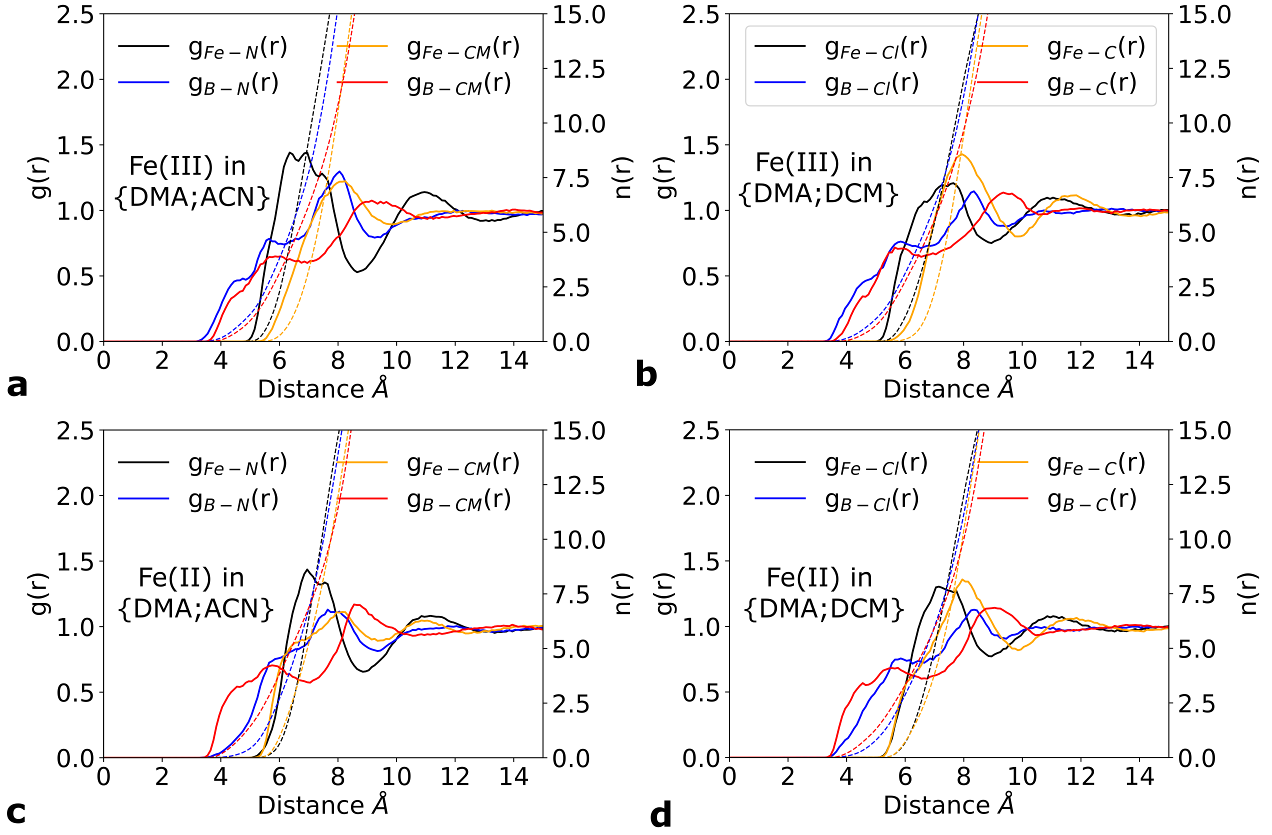

FIG. SI.5: Radial distribution functions ($g(r)$) of FeNHC in solvent mixtures (a) Fe(III)NHC$^+$ in {DMA;ACN}, (b) Fe(III)NHC$^+$ in {DMA;DCM}, (c) Fe(II) in {DMA;ACN}, and (d) Fe(II) in {DMA;DCM}. The full lines correspond to the $g_{Fe-X}(r)$ and $g_{B-X}(r)$ functions for the FeNHC Fe or B atoms, the ACN atoms X = N or CM (C in CH_3) and the DCM atoms X = Cl or C. The dashed lines are the corresponding integrated functions ($n(r)$).

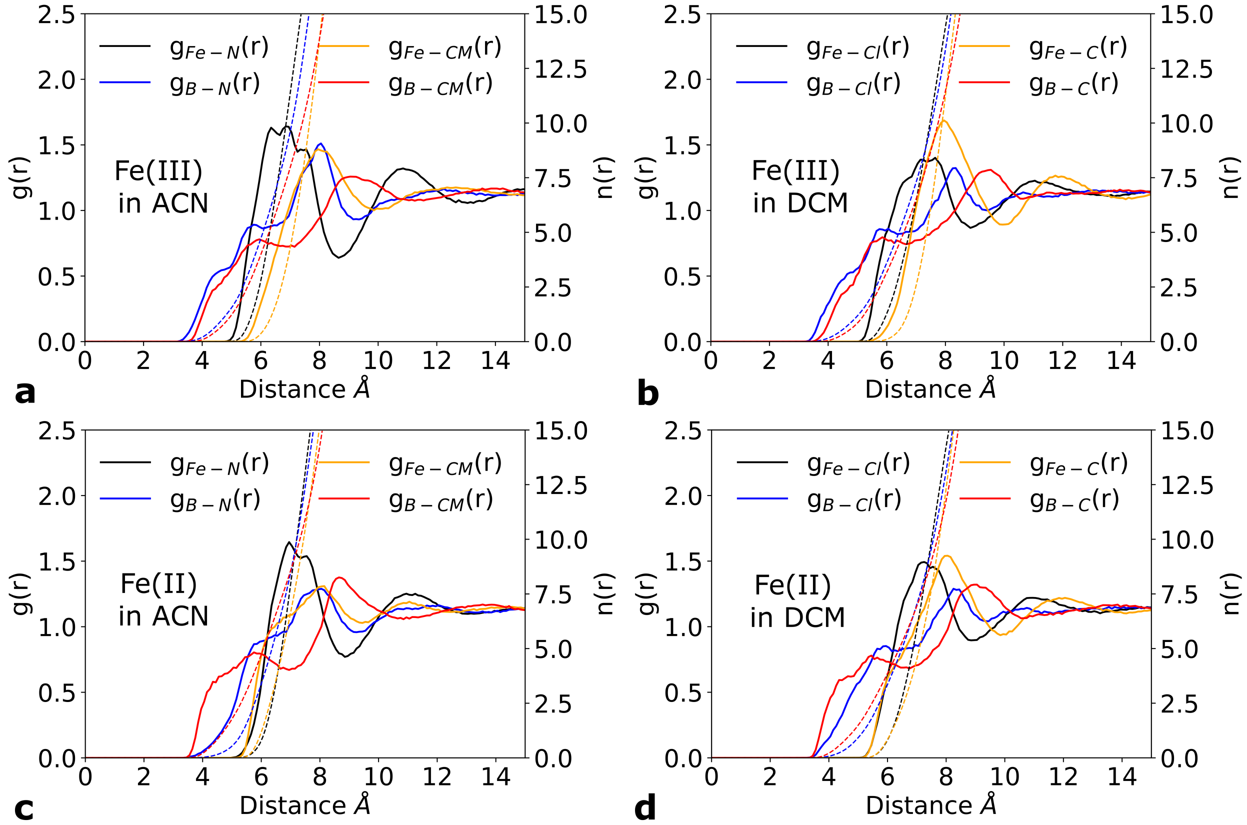

FIG. SI.6: Radial distribution functions ($g(r)$) of (a) Fe(III)NHC$^+$ and DMA in pure ACN, (b) Fe(III)NHC$^+$ and DMA in pure DCM, (c) Fe(II)NHC and DMA in pure ACN and, (d) Fe(II)NHC, and DMA in pure DCM. The full lines correspond to the $g_{Fe-X}(r)$ and $g_{B-X}(r)$ functions for the FeNHC Fe or B atoms and the DMA atoms X = N or C4. The dashed lines are the corresponding integrated functions ($n(r)$).

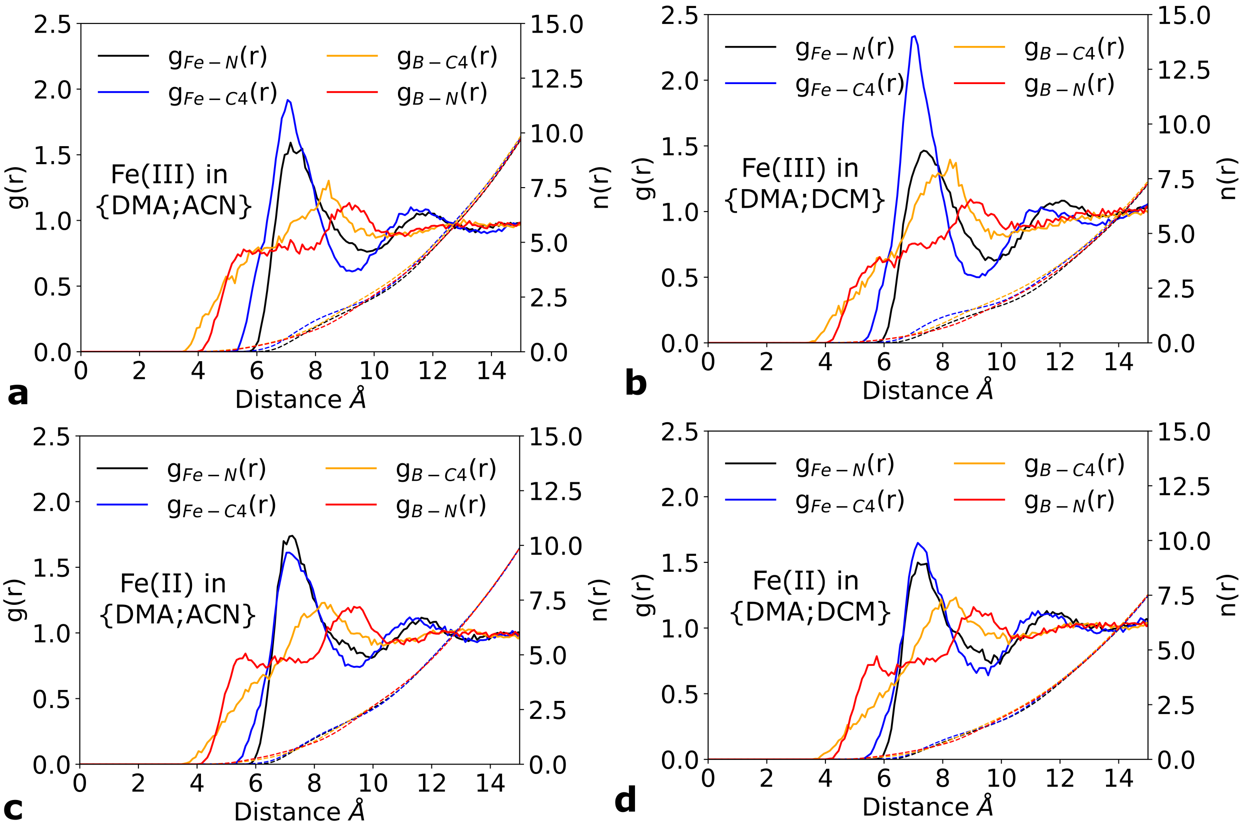

FIG. SI.7: Radial distribution functions ($g(r)$) of (a) Fe(III)NHC$^+$ and DMA in {DMA;ACN}, (b) Fe(III)NHC$^+$ and DMA in {DMA;DCM}, (c) Fe(II)NHC and DMA in {DMA;ACN}, and (d) Fe(II)NHC and DMA in {DMA;DCM}. The full lines correspond to the $g_{Fe-X}(r)$ and $g_{B-X}(r)$ functions for the FeNHC Fe or B atoms and the DMA atoms X = N or C4. The dashed lines are the corresponding integrated functions ($n(r)$).

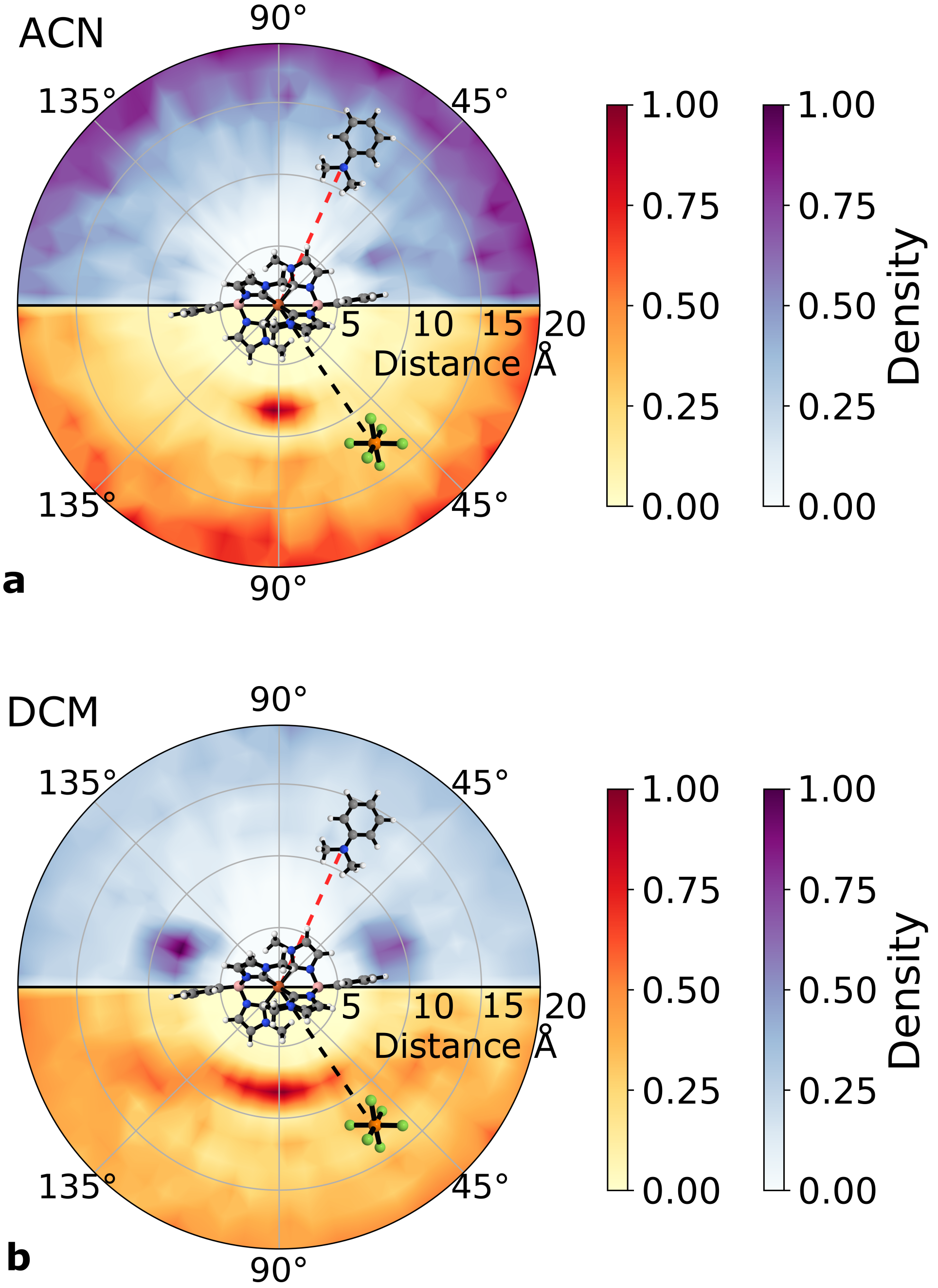

FIG. SI.8: Angular and radial distribution of $DMA^{\bullet+}$ (top blue hemisphere) and PF_6^- (bottom orange hemisphere) with respect to Fe(II)NHC (a) in pure ACN, and (b) in pure DCM. The considered distances and angles are X–Fe(Fe(II)NHC) and B(Fe(II)NHC)–Fe(Fe(II)NHC)–X, where X is either N($DMA^{\bullet+}$) or P(PF_6^-).

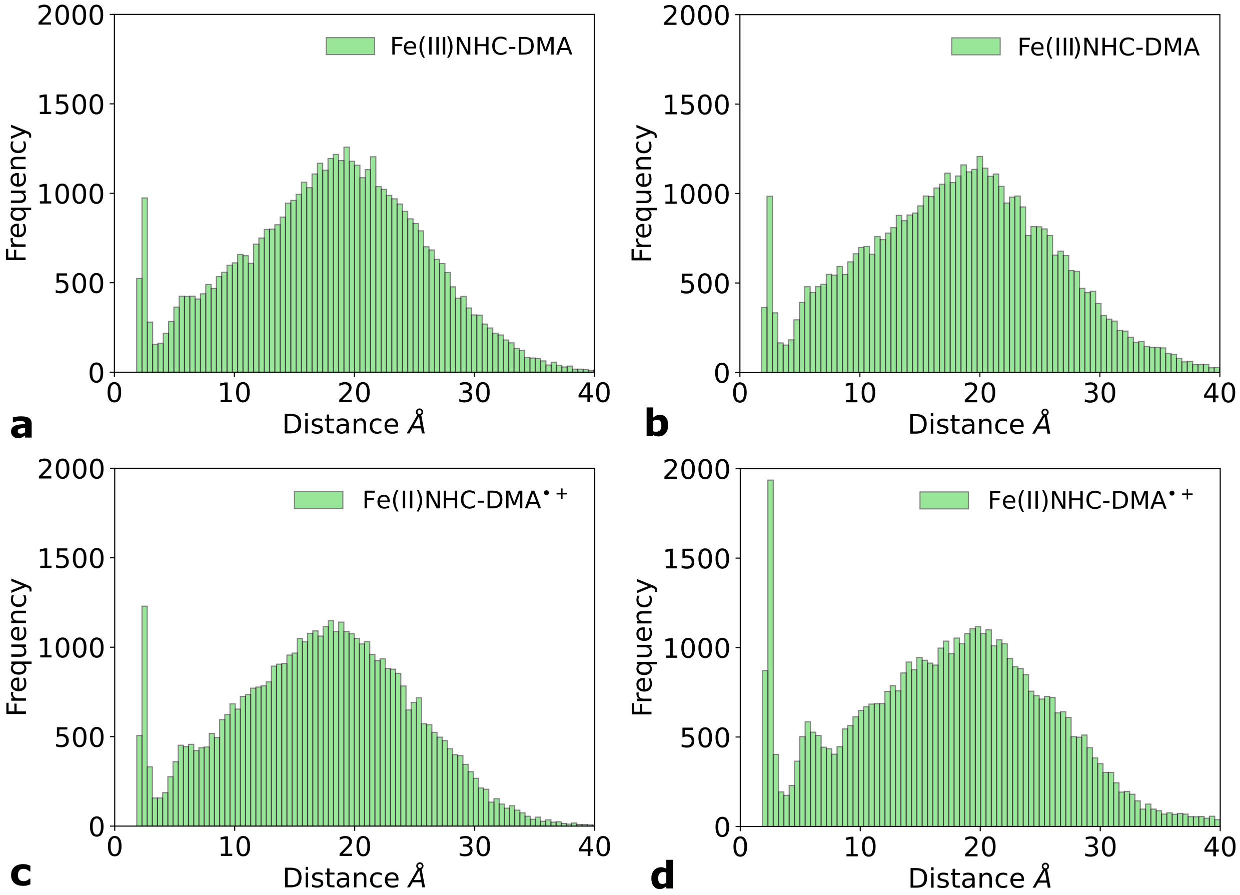

FIG. SI.9: Distribution of minimal distances of (a) Fe(III)NHC^{+} and DMA in {DMA;ACN}, (b) Fe(III)NHC^{+} and DMA in {DMA;DCM}, (c) Fe(II)NHC and DMA$^{\bullet+}$ in {DMA;ACN} and, (d) Fe(II)NHC and DMA$^{\bullet+}$ in {DMA;DCM}.

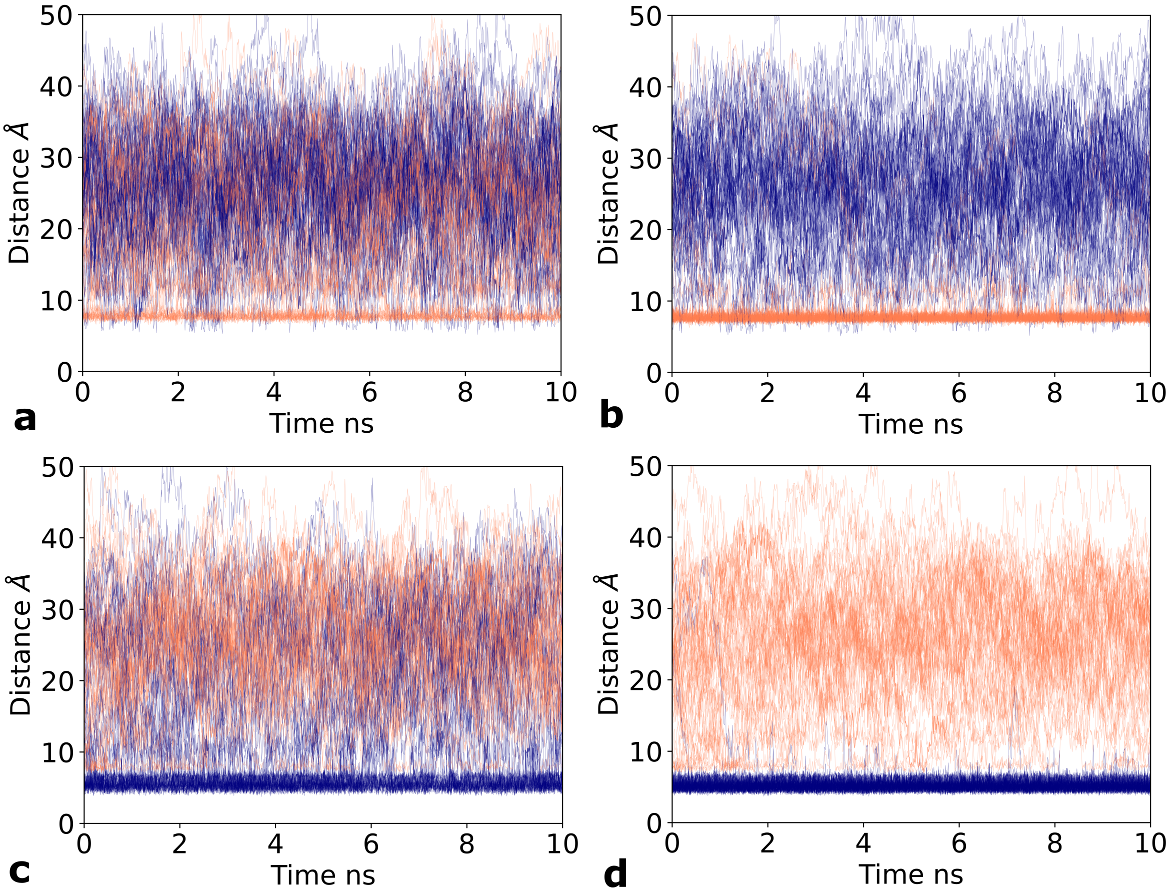

FIG. SI.10: Distances in Å between (a) PF_6^- and $Fe(III)NHC^+$ and DMA in {DMA;ACN}, (b) PF_6^- and $Fe(III)NHC^+$ and DMA in {DMA;DCM}, (c) PF_6^- and Fe(II)NHC and $DMA^{\bullet+}$ in {DMA;ACN} and, (d) PF_6^- and Fe(II)NHC and $DMA^{\bullet+}$ in {DMA;DCM}. Orange trajectories designate PF_6^--FeNHC distances and blue trajectories PF_6^--DMA distances.

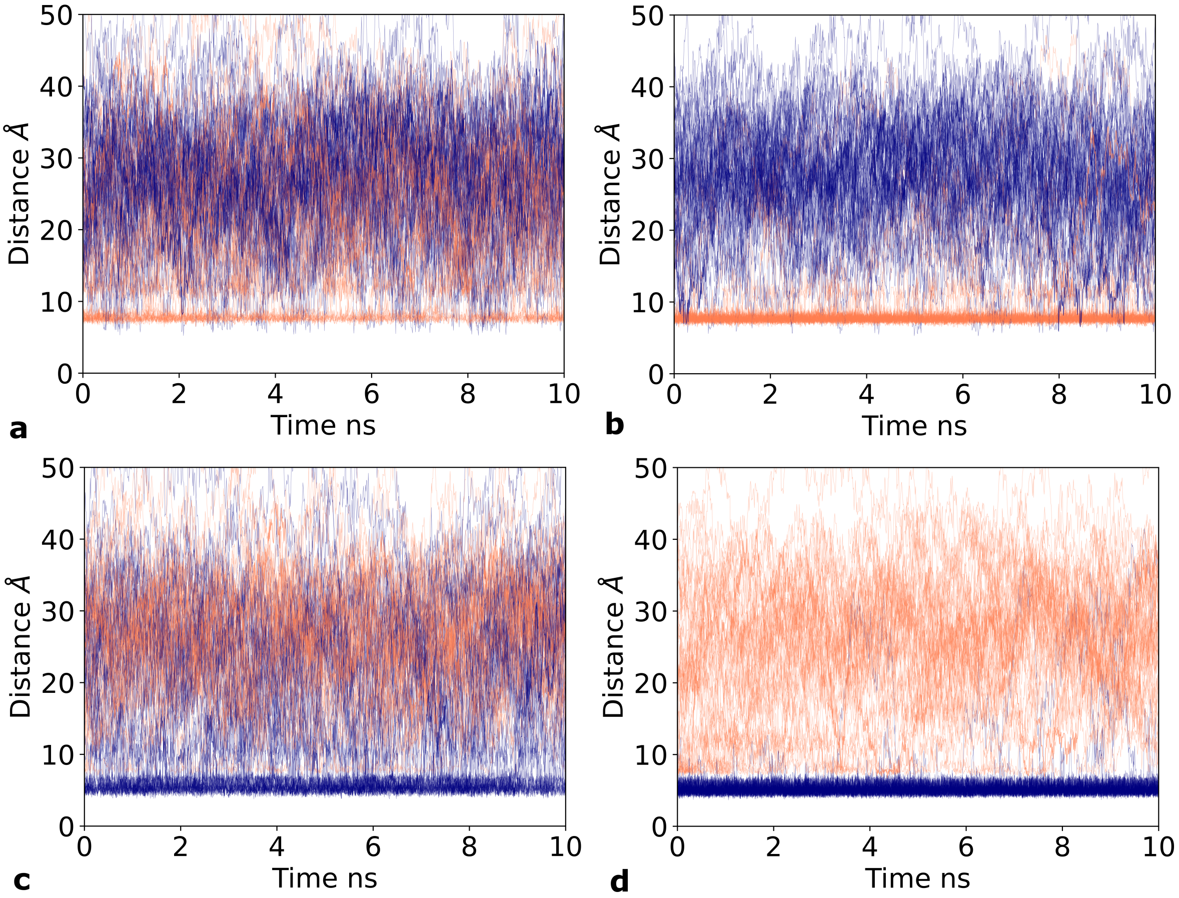

FIG. SI.11: Distances in Å between (a) PF_6^- and Fe(III)NHC$^+$ and DMA in ACN, (b) PF_6^- and Fe(III)NHC$^+$ and DMA in DCM, (c) PF_6^- and Fe(II)NHC and DMA$^{\bullet+}$ in ACN and, (d) PF_6^- and Fe(II)NHC and DMA$^{\bullet+}$ in DCM. Orange trajectories designate PF_6^--FeNHC distances and blue trajectories PF_6^--DMA distances.

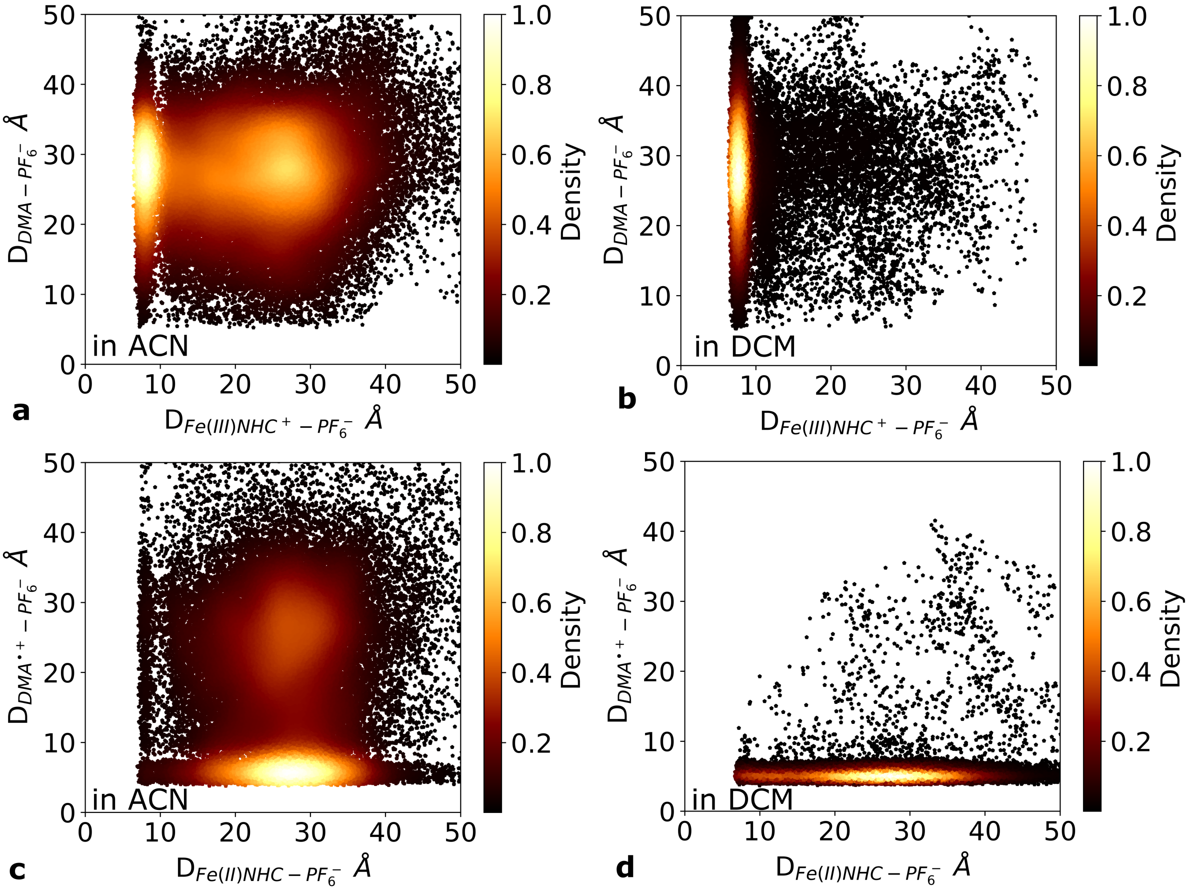

FIG. SI.12: PF_6^- counterion distances with respect to (a) $Fe(III)NHC^+$ and DMA in pure ACN, (b) $Fe(III)NHC^+$ and DMA in pure DCM, (c) Fe(II)NHC and $DMA^{\bullet +}$ in pure ACN, (d) Fe(II)NHC and $DMA^{\bullet +}$ in pure DCM.

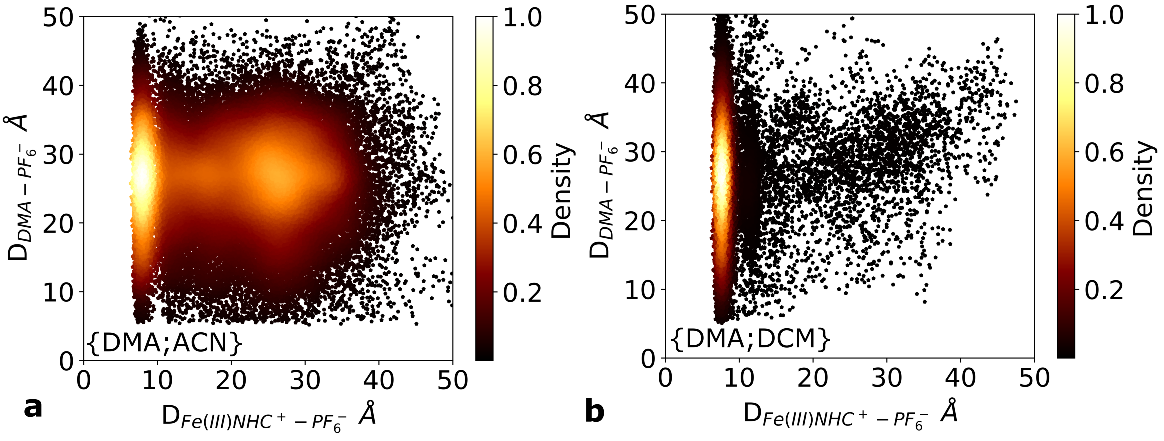

FIG. SI.13: $Fe(III)NHC^+$ and PF_6^- distances with respect to DMA and PF_6^- distances in the charge-separated state for a collection of 500 ns MD simulations in (a) {DMA;ACN}, (b) {DMA;DCM}.

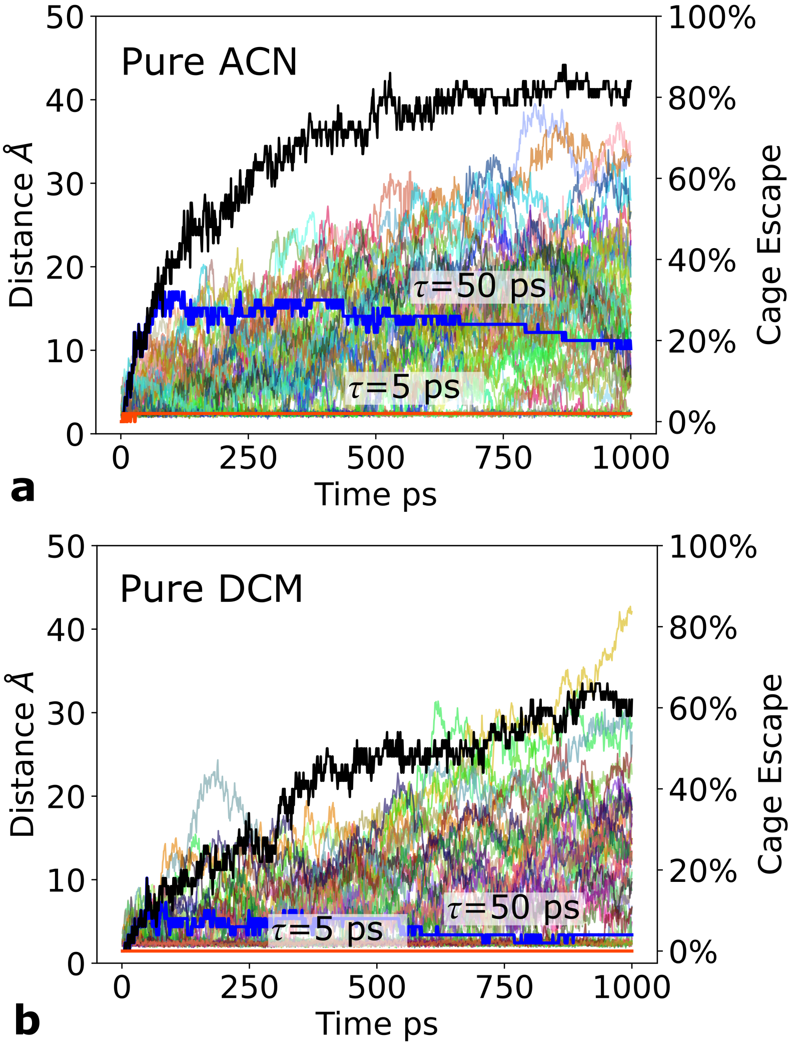

FIG. SI.14: Minimal distance between Fe(II)NHC and $DMA^{\bullet +}$ as a function of time for 50 trajectories (thin color lines) in (a) pure ACN and (b) pure DCM. Calculated cage-escape percentage are displayed in (black) neglecting charge recombination, (blue) 50 ps charge recombination and (orange) 5 ps charge recombination, respectively.

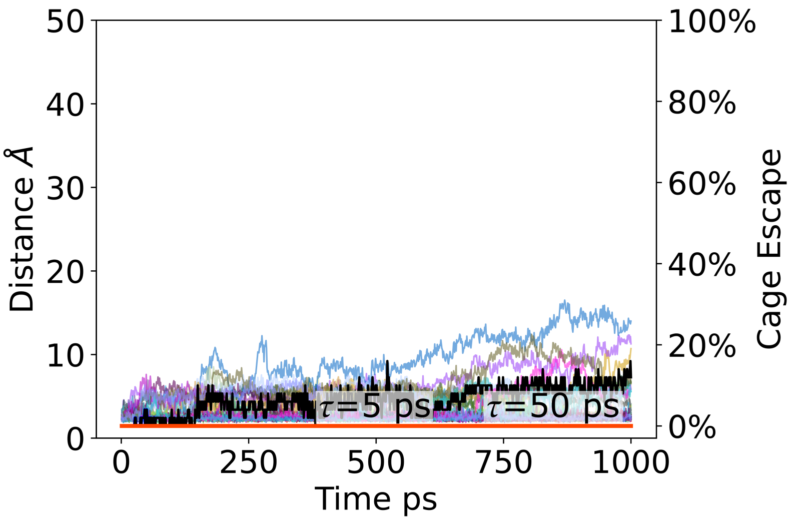

FIG. SI.15: Minimal distance between Fe(II)NHC and $DMA^{\bullet+}$ as a function of time for 50 trajectories (thin color lines) in pure DMA. Calculated cage-escape percentage are displayed in (black) neglecting charge recombination, (blue) 50 ps charge recombination and (orange) 5 ps charge recombination, respectively.

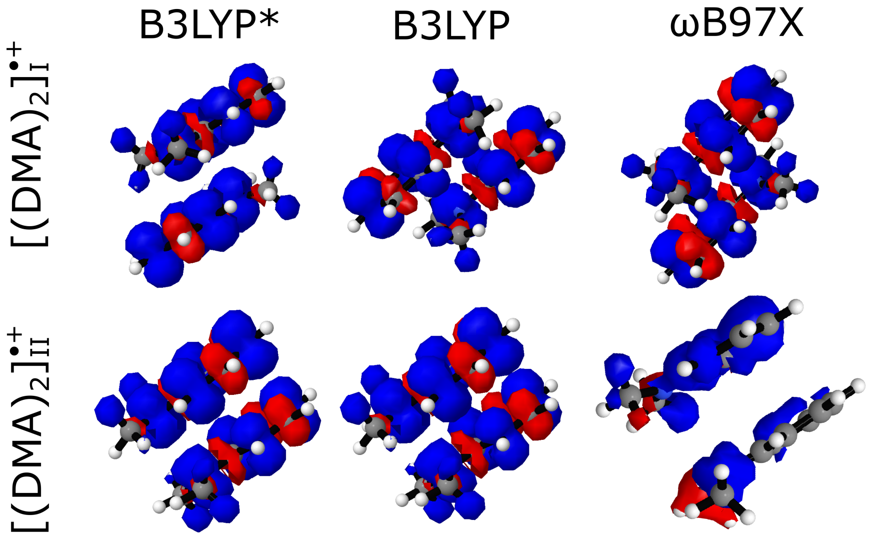

FIG. SI.16: Spin density of dimers $[(DMA)_2]_{I}$ and $[(DMA)_2]_{II}$ calculated with B3LYP*-D3, B3LYP-D3 and ωB97X-D3 in acetonitrile.

System	E_{red}^{calc} / V vs Fc(+1/0) in ACN	E_{red}^{calc} / V vs Fc(+1/0) in DCM
Fe(III/II)	-1.33	-1.37
Fe(III/II)PF_6^-	-1.43	-1.65
Fe(III/II)	0.80	0.76
Fe(III/II)PF_6^-	0.70	0.47
DMA(+1/0)	0.45	0.44
$[(DMA)_2]_I$(+1/0)	0.11	0.08
$[(DMA)_2]_{II}$(+1/0)	-0.03	-0.05
$[(DMA)_3]_I$(+1/0)	-0.04	-0.08
$[(DMA)_3]_{II}$(+1/0)	-0.18	-0.18
[DMA]PF_6(0/-1)	0.35	0.16
$[(DMA)_2]_I PF_6$(0/-1)	-0.19	-0.42
$[(DMA)_2]_{II} PF_6$(0/-1)	-0.12	-0.41
$[(DMA)_3]_I PF_6$(0/-1)	-0.14	-0.36
$[(DMA)_3]_{II} PF_6$(0/-1)	-0.20	-0.63

TABLE SI.3: Calculated reduction potentials of Fe(III/II)NHC, DMA(+1/0), $[(DMA)_n]_I$(+1/0), and $[(DMA)_n]_{II}$(+1/0) (n = 2, 3) with and without the counterion (PF_6^-) in acetonitrile (ACN) and dichloromethane (DCM). All reduction potentials are expressed with respect to the ferrocene redox couple (Fc(+1/0)).

System	B3LYP*	B3LYP	ωB97X
DMA(+1/0)	0.45	0.43	0.38
$[(DMA)_2]_I$(+1/0)	0.11	0.14	0.26
$[(DMA)_2]_{II}$(+1/0)	-0.03	0.01	0.12
[DMA]PF_6(0/-1)	0.35	0.32	0.24
$[(DMA)_2]_I PF_6$(0/-1)	-0.19	-0.17	0.07
$[(DMA)_2]_{II} PF_6$(0/-1)	-0.12	0.02	-0.06

TABLE SI.4: Calculated reduction potentials (in V vs. Fc(+1/0)) of DMA, $[(DMA)_2]_I$, $[(DMA)_2]_{II}$, [DMA]PF_6^-, $[(DMA)_2]_I PF_6^-$ and $[(DMA)_2]_{II} PF_6^-$ with B3LYP*-D3, B3LYP-D3 and ωB97X-D3 in acetonitrile (ACN).

System	B3LYP*	B3LYP	ωB97X
DMA(+1/0)	0.44	0.43	0.38
$[(DMA)_2]_I$(+1/0)	0.08	0.12	0.24
$[(DMA)_2]_{II}$(+1/0)	-0.05	-0.02	0.15
[DMA]PF_6(0/-1)	0.16	0.10	0.00
$[(DMA)_2]_I PF_6$(0/-1)	-0.42	-0.41	-0.32
$[(DMA)_2]_{II} PF_6$(0/-1)	-0.41	-0.18	-0.31

TABLE SI.5: Calculated reduction potentials (in V vs. Fc(+1/0)) of DMA, $[(DMA)_2]_I$, $[(DMA)_2]_{II}$, [DMA]PF_6^-, $[(DMA)_2]_I PF_6^-$ and $[(DMA)_2]_{II} PF_6^-$ with B3LYP*-D3, B3LYP-D3 and ωB97X-D3 in dichloromethane (DCM).

System	ΔG^{calc}_{bind} kcal/mol in ACN	ΔG^{calc}_{bind} kcal/mol in DCM
$[(DMA)_2]_I$	8.18	7.89
$[(DMA)_2]_I^{\bullet+}$	0.78	-0.08
$[(DMA)_2]_{II}$	9.45	9.31
$[(DMA)_2]_{II}^{\bullet+}$	-0.98	-1.38
$[(DMA)_3]_I$	17.57	17.01
$[(DMA)_3]_I^{\bullet+}$	6.70	6.28
$[(DMA)_3]_{II}$	18.75	17.58
$[(DMA)_3]_{II}^{\bullet+}$	4.56	4.32
$[DMA]PF_6^-$	9.72	7.68
$[DMA]^{\bullet+}PF_6^-$	7.06	2.84
$[(DMA)_2]_I PF_6^-$	18.59	17.91
$[(DMA)_2]_I^{\bullet+}PF_6^-$	3.54	-1.38
$[(DMA)_2]_{II} PF_6^-$	19.39	18.78
$[(DMA)_2]_{II}^{\bullet+}PF_6^-$	7.04	0.85
$[(DMA)_3]_I PF_6^-$	26.67	26.21
$[(DMA)_3]_I^{\bullet+}PF_6^-$	13.80	7.68
$[(DMA)_3]_{II} PF_6^-$	27.88	27.10
$[(DMA)_3]_{II}^{\bullet+}PF_6^-$	15.47	8.35

TABLE SI.6: Calculated binding free energies (ΔG^{calc}_{bind}) for the formation of neutral and radical-cation dimers and trimers $[(DMA)_n]_I$, and $[(DMA)_n]_{II}$ ($n = 2, 3$) with and without the counterion PF_6^- in acetonitrile (ACN) and dichloromethane (DCM).

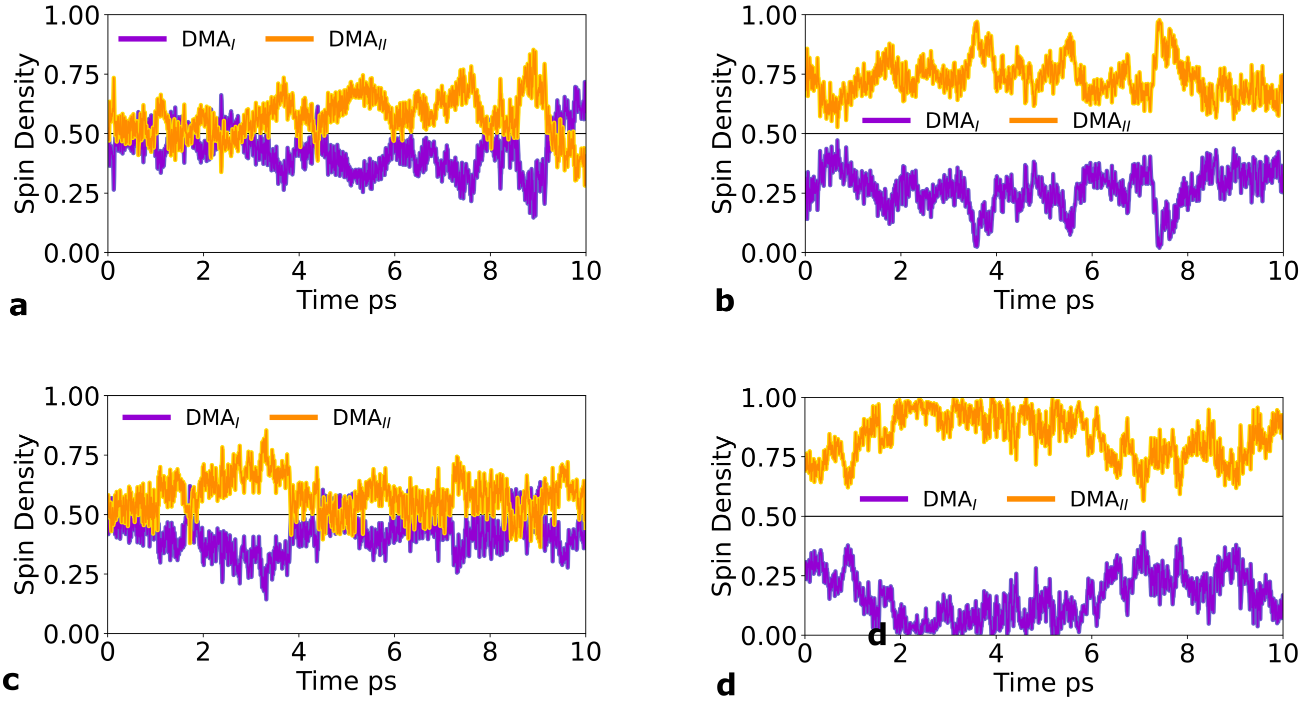

FIG. SI.17: (a–d) Spin density evolution of the radical-cation dimer (the spin on each monomer is shown in violet and orange, respectively) during 10 ps of QM/MM MD simulation number (a) three, (b) four, (c) five, and (d) six.

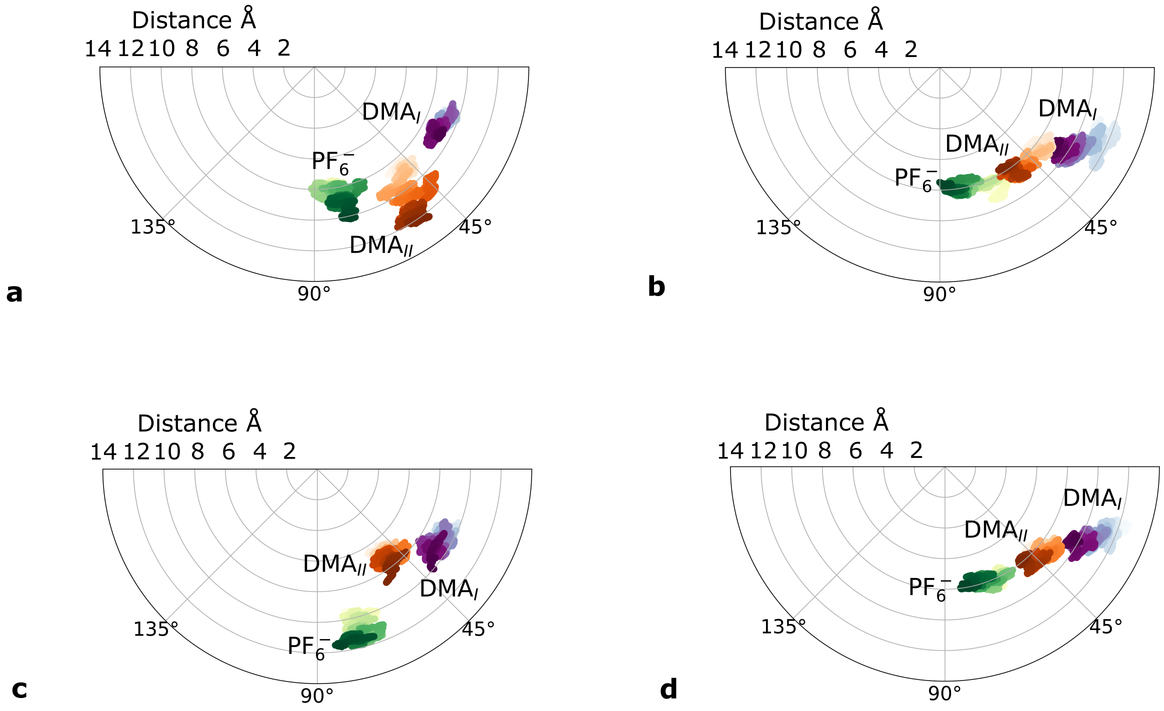

FIG. SI.18: (a–d) Angular and radial trajectories of monomer DMA_I, monomer DMA_{II}, and PF_6^- with respect to Fe(II)NHC during the 10 ps of QM/MM MD simulation number (a) three, (b) four, (c) five, and (d) six. The distances and angles are calculated for the Fe(FeNHC), N(DMA_I), N(DMA_{II}), and P(PF_6^-) atoms. The color gradients illustrate the time progression (light in the start and dark at the end).

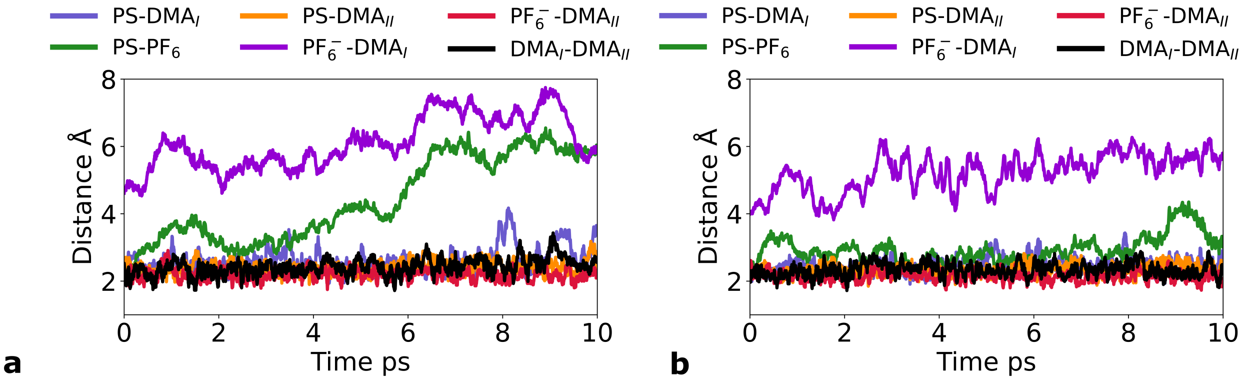

FIG. SI.19: Minimal distances during 10 ps of the QM/MM MD simulation number (a) one, and (b) two between (blue) photosensitizer and monomer DMA_I, (orange) photosensitizer and monomer DMA_{II}, (green) photosensitizer and counterion, (purple) counterion and monomer DMA_I, (red) counterion and monomer DMA_{II}, and (black) monomer DMA_I and monomer DMA_{II}.

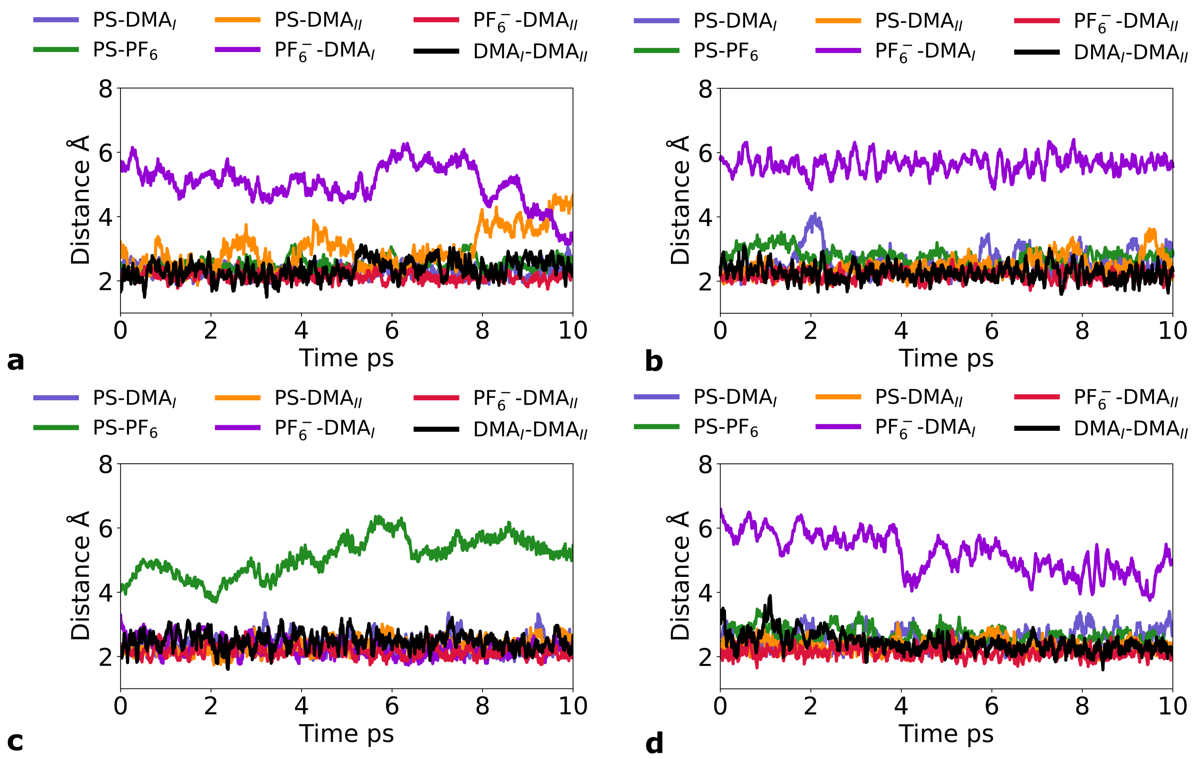

FIG. SI.20: Minimal distances during 10 ps of the QM/MM MD simulation number (a) three, (b) four, (c) five, and (d) six between (blue) photosensitizer and monomer DMA$_I$, (orange) photosensitizer and monomer DMA$_{II}$, (green) photosensitizer and counterion, (purple) counterion and monomer DMA$_I$, (red) counterion and monomer DMA$_{II}$, and (black) monomer DMA$_I$ and monomer DMA$_{II}$.

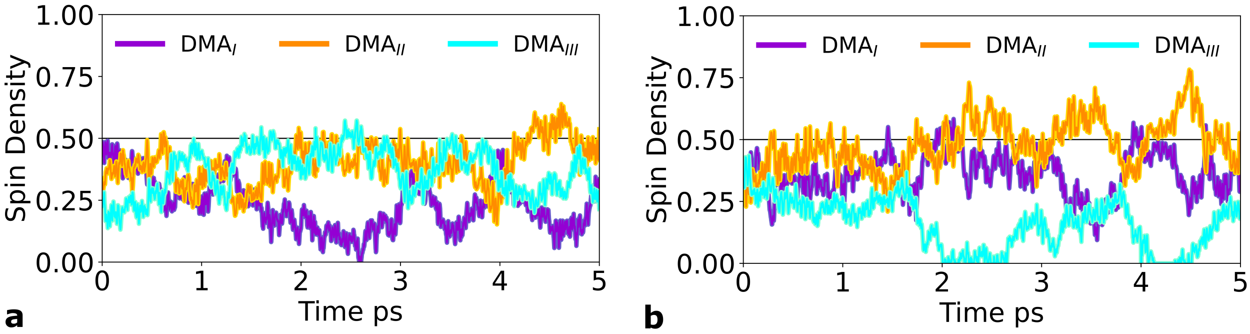

FIG. SI.21: Spin density evolution of the radical-cation including three DMA monomers (the spin on each monomer is shown in violet, orange and blue, respectively) and PF_6^- (green) during 5 ps of QM/MM MD simulation number (a) three and, (b) five.

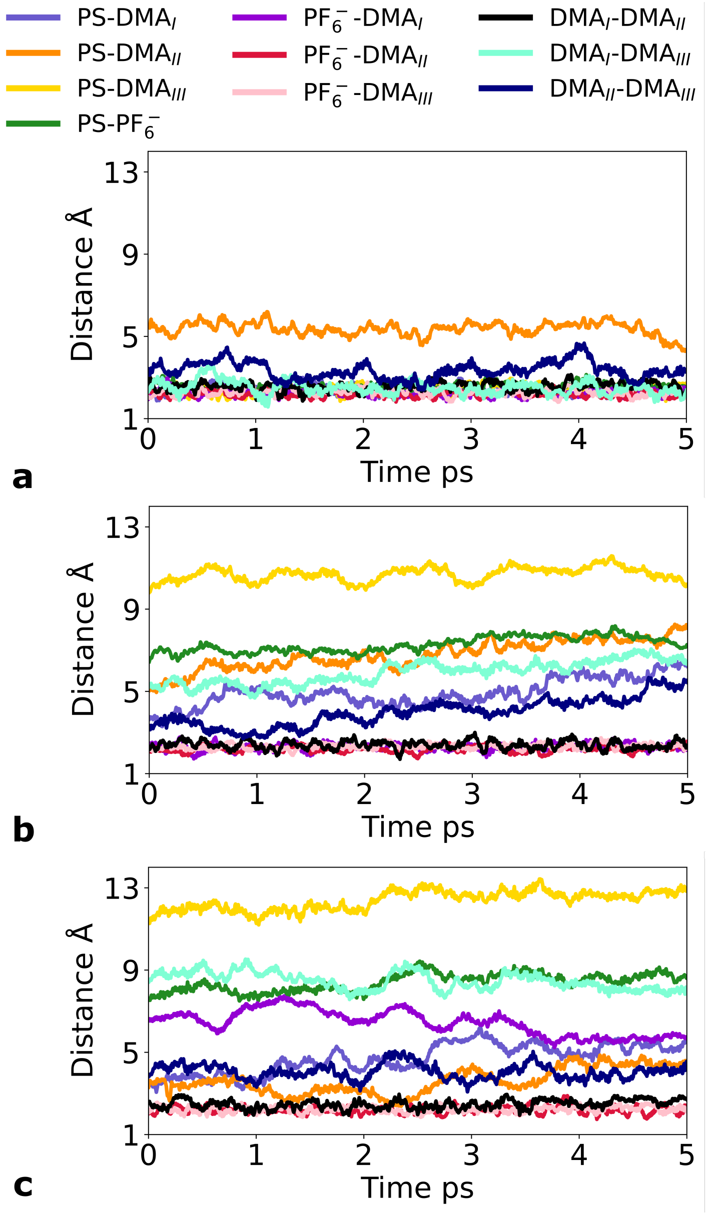

FIG. SI.22: Minimal distances during 5 ps of QM/MM MD simulation number (a) one, (b) three, and (c) five between (blue) photosensitizer and monomer DMA$_I$, (orange) photosensitizer and monomer DMA$_{II}$, (yellow) photosensitizer and monomer DMA$_{III}$, (green) photosensitizer and counterion, (purple) counterion and monomer DMA$_I$, (red) counterion and monomer DMA$_{II}$, (pink) counterion and monomer DMA$_{III}$, (black) monomer DMA$_I$ and monomer DMA$_{II}$, (light green) monomer DMA$_I$ and monomer DMA$_{III}$, and (navy) monomer DMA$_{II}$ and monomer DMA$_{III}$.

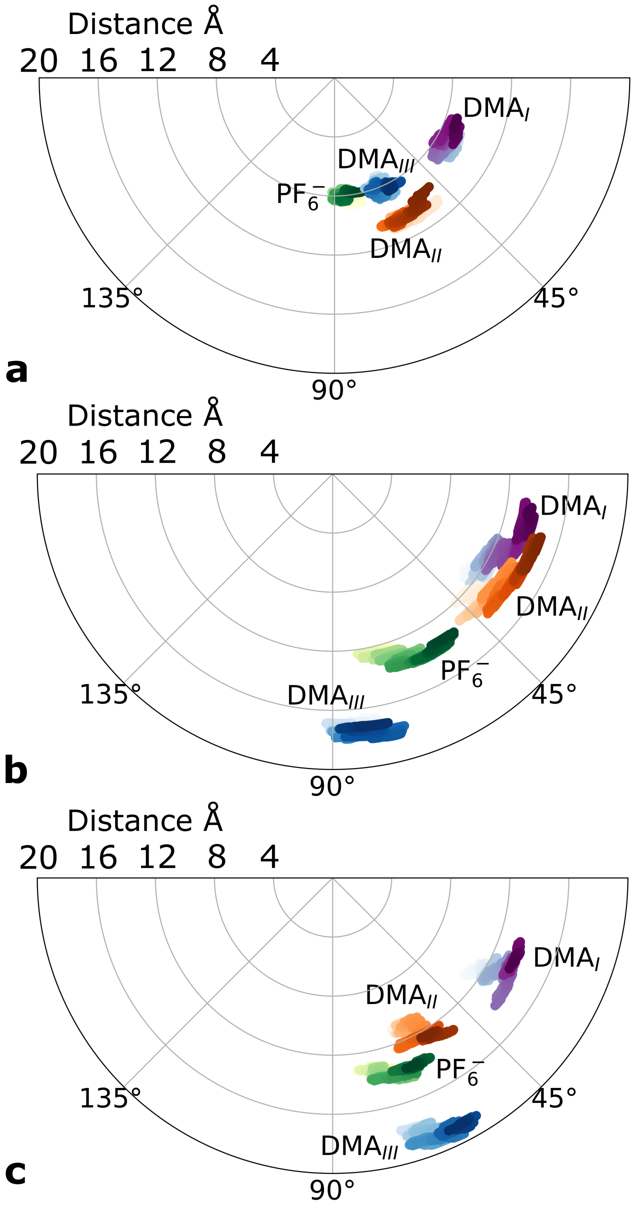

FIG. SI.23: (a–c) Angular and radial trajectories of monomer DMA_I, monomer DMA_{II}, monomer DMA_{III}, and PF_6^- with respect to Fe(II)NHC during the 5 ps of QM/MM MD simulation number (a) one, (b) three and (c) five. The distances and angles are calculated for the Fe(FeNHC), N(DMA_I), N(DMA_{II}), N(DMA_{III}), and P(PF_6^-) atoms. The color gradients illustrate the time progression (light in the start and dark at the end).
